# The evaluation of JAK inhibitors on effect and safety in alopecia areata: a systematic review and meta-analysis of 2018 patients

**DOI:** 10.3389/fimmu.2023.1195858

**Published:** 2023-06-02

**Authors:** Mei-qi Mao, Yu-xin Ding, Jing Jing, Zhen-wei Tang, Yu-jie Miao, Xiao-shuang Yang, Yu-hong Chen, Sheng-zhao Chen, Xian-jie Wu, Zhong-fa Lu

**Affiliations:** Department of Dermatology, Second Affiliated Hospital, Zhejiang University School of Medicine, Hangzhou, China

**Keywords:** JAK/STAT (janus kinase/signal transducer and activator of transcription), JAK inhibitors, alopecia areata, immune-mediated diseases, meta-analysis

## Abstract

**Background:**

JAK inhibitors treat various autoimmune diseases, but an updated systematic review in treating alopecia areata is currently lacking.

**Objective:**

Evaluate the specific efficacy and safety of JAK inhibitors in alopecia areata by systematic review and meta-analysis.

**Methods:**

Eligible studies in PubMed, Embase, Web of Science, and Clinical Trials up to May 30, 2022, were searched. We enrolled in randomized controlled trials and observational studies of applying JAK inhibitors in alopecia areata.

**Results:**

6 randomized controlled trials with 1455 patients exhibited SALT_50_ (odd ratio [OR], 5.08; 95% confidence interval [CI], 3.49-7.38), SALT_90_ (OR, 7.40; 95% CI, 4.34-12.67) and change in SALT score (weighted mean difference [WSD], 5.55; 95% CI, 2.60-8.50) compared to the placebo. The proportion of 26 observational studies with 563 patients of SALT_5_ was 0.71(95% CI, 0.65-0.78), SALT_50_ was 0.54(95% CI 0.46-0.63), SALT_90_ was 0.33(95% CI, 0.24-0.42), and SALT score (WSD, -2.18; 95% CI, -3.12 to -1.23) compared with baseline. Any adverse effects occurred in 921 of 1508 patients; a total of 30 patients discontinued the trial owing to adverse reactions.

**Limitations:**

Few randomized controlled trials met the inclusion criteria and insufficiency of eligible data.

**Conclusion:**

JAK inhibitors are effective in alopecia areata, although associated with an increased risk.

## Introduction

Alopecia areata (AA) is a commonly occurring autoimmune disorder, with a prevalence rate of 2% in the United States (1). Persistent AA and its variants can lead to significant scalp hair loss, adversely impacting the patient’s quality of life and psychological well-being (2). Currently, there are no available drugs for permanent AA treatment. Clinical drug regimens mainly rely on intra-lesion or systemic corticosteroids, minoxidil, and methotrexate. However, patients with moderate to severe alopecia areata (SALT score≥50%), especially those with alopecia totalis or universalis, require more effective, better tolerated, and safer alternative drugs (3–5).

AA is a degenerative disease that affects hair follicles and is characterized by inflammatory cell infiltration around lesion follicles. The clinical manifestations include sudden, circular patchy hair loss on the scalp and other areas such as eyebrows, eyelashes, beard, and body hair, along with dotted depression of finger/toe nails (6). Some oral JAK inhibitors (JAKi) have been approved by the FDA for the treatment of autoimmune diseases such as rheumatoid arthritis, psoriasis, and allergic dermatitis; however, as of June 2022, only Baricitinib had garnered approval from FDA (7–10). Pfizer’s new oral JAKi PF-06651600 and Concert Pharmaceuticals’ CTP-543 and topical ATI-502 have received ‘Fast Track’ from the FDA and completed Phase III RCTs to generate efficacy and safety data for future JAKi applications in AA. Additional studies are required to establish their effectiveness and safety. To that end, we undertook a systematic review and meta-analysis of published RCTs and OSs to evaluate the effectiveness and safety of JAKi in AA treatment.

## Methods

This investigation was conducted in adherence with the Preferred Reporting Items for Systematic Reviews and Meta-analyses (PRISMA) guidelines (11) (**Supplemental Table 1** in Supplement material). The primary design was registered on PROSPERO (CRD42022334326).

Our study examined the impact of pre- and post-JAKi therapies on AA patients’ outcomes. The primary evaluation was the variation in Severity in Alopecia Tool (SALT) score, comparing the experimental group to the placebo group in the randomized controlled trials (RCTs), and post-treatment values to the baseline in the observation studies (OSs). Secondary measurements included the proportion of patients achieving improvements of 5%, 50%, and 90% in the SALT score (SALT_5_, SALT_50_, SALT_90_) and adverse event rates.

### Data source and search strategy

The study utilized several pertinent databases, including PubMed, Embase, Web of Science, and Clinical Trials, to gather relevant data up to May 30, 2022. The search was specifically focused on the keywords “Alopecia areata” and “JAK inhibitor,” or “Ruxolitinib or Tofacitinib or Baricitinib.”

Furthermore, we examined the reference lists of all the retrieved articles to identify studies that possibly provided suitable information. The assessment was performed using inclusion and exclusion criteria. Additionally, we have presented the complete search strategy in **Supplemental Table 2**.

### Study selection

Eligibility criteria consisted of (I) Studied patients with AA were pathological examination confirmed. (II) Change in SALT was used as an indicator. (III) Inclusion of case reports, case series, cohort studies, or clinical trials of patients with AA and JAKi. To ensure accuracy, the titles and abstracts of selected articles were screened independently by two authors, Mei-qi Mao and Jing Jing. Upon finding insufficient information in the abstracts, a full-text review was performed.

Exclusive criteria included (I) Review, meta-analysis, systematic evaluation, meeting abstracts, and case reports (less than 3 cases). (II) Clinical trials of non-oral JAKi. (III) Clinical trials to detect the effect of JAKi application on eyebrows and eyelashes. (IV) Studies were not written in Chinese or English. (V) Studies had duplicated data or repeat analyses. (VI) Studies with insufficient data. These criteria were put in place to ensure the reliability and efficiency of the study (refer to **Supplemental Figure 1** for more details).

### Quality assessment

Two authors (Mei-qi Mao and Jing Jing) performed the quality assessment independently. Any disagreements were discussed with the third author (Yu-xin Ding) and resolved by consensus. The RCTs were assessed using the Cochrane Collaboration Risk-of-bias Instrument (12). OSs quality assessment were based on the Joanna Briggs Institute Critical Appraisal tools (13).

### Statistical analysis

The weighted mean difference (WMD) with a 95% confidence interval (CI) was used to estimate the continuous data, such as the SALT score improvement pre- and post-treatment. For dichotomous variables such as rate of SALT_5_, SALT_50_, SALT_90_ and adverse events, we used pooled odds ratios (ORs) with 95% CIs. Random effects analysis was applied using Dorsmanin and Laird method (14). Subgroup analysis dealt with heterogeneity, and *P*<0.05 was considered statistically significant. Publication bias was assessed *via* funnel plots and Begg’s plots, with *P*<0.1 indicating asymmetric funnel plots. All the described analyses were performed with Stata17 (StataCorp, Texas, USA).

## Result

### Search results and trial characteristics

A final total of 32 articles were included through electronic database searching, including six controlled clinical trials (including 1455 patients) (15–20) and 26 case reports or case series (including 563 patients) (**Supplementary Table 3**) (21–46).

### Quality assessment

The RCTs were determined to be low risk in the assessment domain, as indicated in **Supplemental Figure 2**. However, observation studies assessments revealed that some studies exhibited high risks due to inadequate control of confounding factors, as depicted in **Supplemental Figure 3**.

### JAKi ameliorates SALT scores

**Figure 1:**
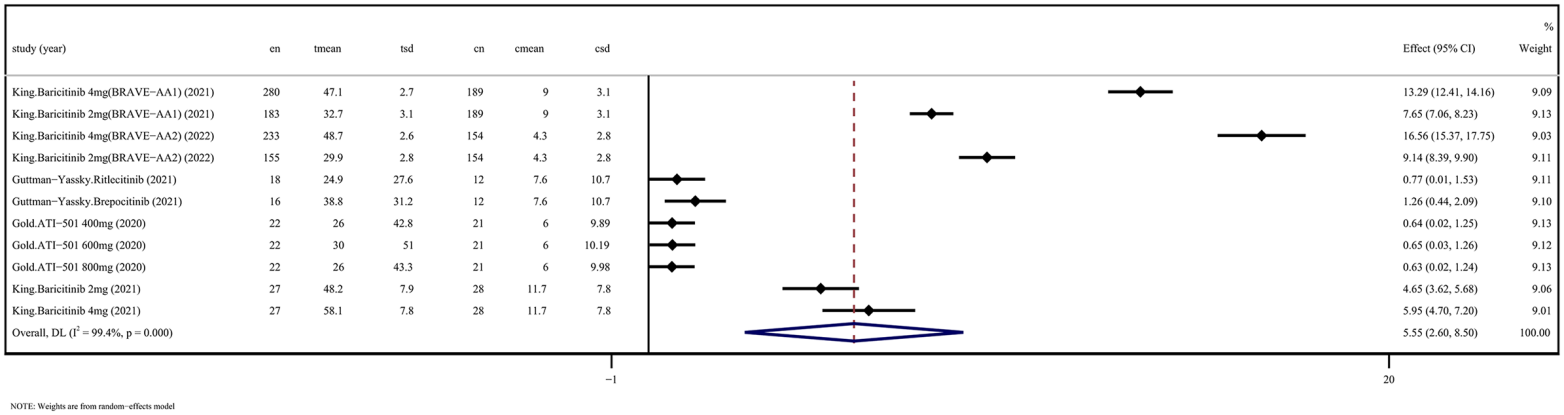
JAKi efficacy compared to placebo on SALT score improvement in randomized controlled trials. CI, Confidence interval; JAKi, Janus kinase inhibitor; SALT, Severity in Alopecia Tool.

**Figure 2:**
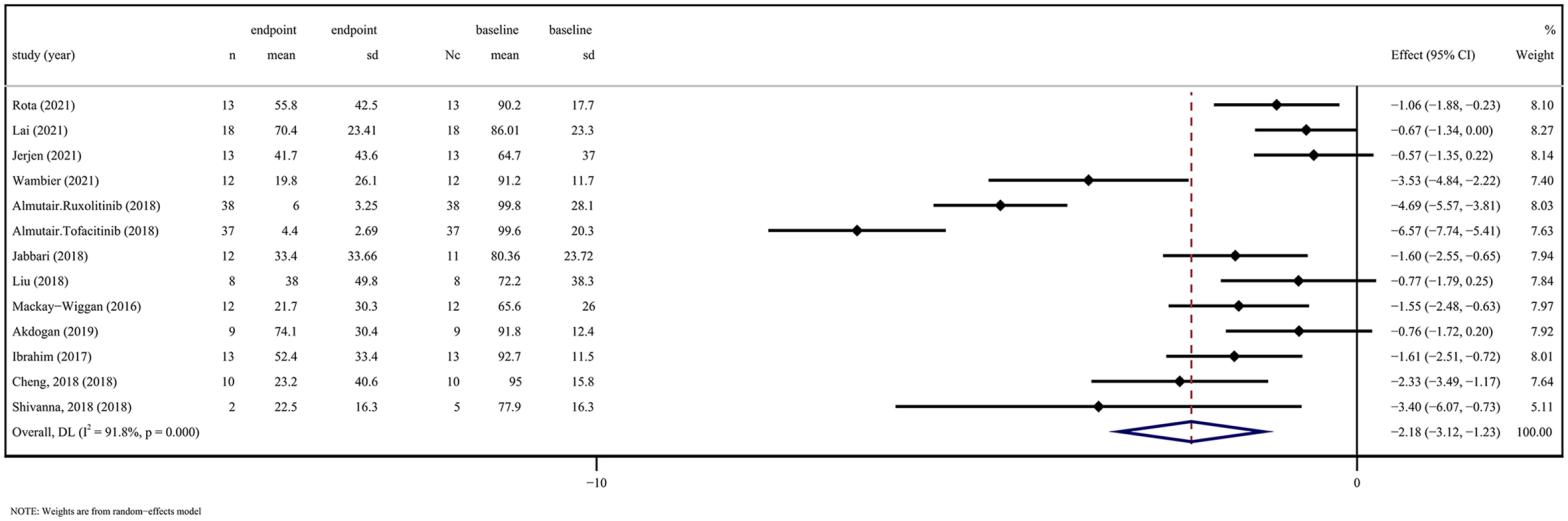
JAK inhibitors efficacy compared to baseline on SALT score in observation studies. CI, Confidence interval; JAK, Janus kinase; SALT, Severity in Alopecia Tool.

5 RCTs assessed changes in SALT scores after application of JAKi compared with changes in placebo treatment (WSD 5.55 [95% CI 2.60-8.50] *P* = 0.000, I^2 ^= 99.4%) (**Figure 1**). 12 OSs assessed SALT scores after administration of JAKi compared with the baseline (WSD -2.18 [95% CI -3.12 to -1.23] *P* = 0.000, I^2 ^= 91.8%) (**Figure 2**). These studies unequivocally indicated the effectiveness of JAKi. For further insights, please refer to **Supplementary Table 4** for the subgroup analysis.

### JAKi boosts the proportion of patients achieving SALT_5_, SALT_50_ and SALT_90_

According to the results of the RCTs, the JAKi treatment group demonstrated statistically significant improvement compared to the placebo group in achieving SALT_50_ and SALT_90_. Specifically, the OR for SALT_50_ was 5.08(95% CI, 3.49-7.38; *P* = 0.879, I^2 ^= 0.0%), and for SALT_90_, it was 7.40(95% CI, 4.34-12.67; *P* = 0.452, I^2 ^= 0.0%), as indicated in **Supplemental Figure 4**. Regarding OS, our evaluation of the proportion of SALT score improvement revealed that the JAKi treatment had a positive effect. The proportion of SALT_5_ was 0.71 ([95% CI 0.65-0.78], *P* = 0.021, I^2 ^= 46.7%), SALT_50_ was 0.54 ([95% CI 0.46-0.63], *P* = 0.000, I^2 ^= 75.7%) and SALT_90_ was 0.33 ([95% CI 0.24- 0.42], *P* = 0.000, I^2 ^= 71.1%) (**Supplemental Figure 5**). These findings demonstrate that JAKi treatment increases the percentage of AA patients with SALT score improvement up to a specified percentage. For further details regarding subgroup analysis, please refer to **Supplementary Tables 4–7**.

### Safety analysis of JAKi for AA

**Supplemental Table 8** presents an overview of safety indicators frequently evaluated in clinical trials. Of the 1508 patients who participated in 20 studies, adverse effects were reported in 921 or approximately 61.1% of cases. Of 1776 patients across 27 studies, only 31 or roughly 1.7% developed severe adverse reactions. Further, the trial witnessed the discontinuation of 30 patients due to adverse reactions. The rates of common adverse reactions, including total infection, abnormal laboratory indicators, neurological symptoms, gastrointestinal reactions, and skin symptoms, are outlined in **Supplemental Figures 6–10**. Notably, no instances of malignancy or tuberculosis arose during the trial period. **Supplementary Tables 9–13** can provide further insights into subgroup analysis.

### Publication bias and inconsistency

The study’s publication bias is evident in both the funnel plot and Begg’s plots. The results of the Begg’s Test demonstrated no significant publication bias for RCTs (*P*=0.961>0.05) and OSs (*P*=0.652>0.05), as indicated in **Supplemental Figures 11–13**.

## Discussion

The current systematic review reveals that the analysis of RCTs and OSs outcome indicators presents statistically significant evidence that JAKi significantly promotes hair regrowth in patients with AA. Notably, subgroup analysis demonstrates a positive effect on hair regrowth for female patients, with Ruxolitinib showing superiority over tofacitinib, and higher Tofacitinib daily dosing having a more significant impact on efficacy. Concerning safety assessment, the observed frequency of adverse reactions was 61.1%, while severe adverse reactions were negligible, with a mere 1.7% of all documented cases. Notably, 30 patients withdrew from the trial due to adverse reactions. Finally, the review was conducted to determine frequencies of typical mild adverse events; the outcomes fetched were within tolerable ranges.

Currently, first-line treatments for AA consist of a range of drugs including compound glycopyrrolate, topical or systemic steroid hormones, topical or oral minoxidil. Second-line treatment options, such as JAK inhibitors, diphenylcyclopropenone, and immunosuppressants, are typically reserved for severe cases where the lesion areas exceed 50%. It is important to note that presently, only Baricitinib has received FDA approval for treating AA (47). While fast-acting, glucocorticoids can be associated with significant pain and severe side effects due to their non-selective targeting of immune cells (48). Off-label immunosuppressive drugs like methotrexate and cyclosporine A constitute systemic therapies, with the latter being recommended in combination with steroids to manage intractable AA cases (49, 50). In summary, given the associated risks, systemic therapies should be reserved for patients with refractory AA and significant psychosocial stress. Low-risk local management represents a reasonable option for instances where AA is localized or limited in extent, particularly in the long run (51).

The JAK/STAT signaling pathway serves as a crucial intersection for numerous inflammatory factors, with JAK inhibitors offering the advantages of swift onset, notable effectiveness, and minimal adverse effects (52). JAKi application has been shown to promote hair regrowth in various areas such as the eyelashes, eyebrows, beards on the face, arms, legs, armpits, and groin region (53). Laboratory findings demonstrated increased hair keratin levels and reduced perifollicular T-lymphocyte infiltration following JAKi therapy, with treatment-related downregulation of inflammatory markers evident in gene expression profiles (17, 43).While JAKi’s effectiveness in treating alopecia areata may not exceed that of systemic glucocorticoids in present evidence, their targeted immunosuppressive properties may offer a safer alternative to long-term systemic therapy (54). Currently, JAKi usage in clinical practice is supplemented with other drugs, such as topical hormones and minoxidil, resulting in significant gains in refractory AA therapy (23). Given that approved JAKi are metabolized by CYP3A4 enzymes, caution must be taken when used in conjunction with inducers such as rifampin or inhibitors such as ketoconazole (48). However, due to data limitations, our study did not evaluate the impact of concomitant therapy with multiple agents.

This meta-analysis has several limitations that should be acknowledged. Firstly, despite our effort to include all randomized controlled trials assessing oral JAK inhibitors, the number of placebo-controlled studies with full reporting is scarce, which precluded us from conducting subgroup and sensitivity analyses. Additionally, this systematic review encompassed many case series and reports characterized by small scale, low quality, high risk of bias, and limited statistical power analysis. Secondly, the present study, along with most of the clinical trials examined, encompassed severe alopecia areata, comprising alopecia totalis and alopecia universalis. Patients with alopecia universalis and alopecia totalis exhibited superior outcomes in response to JAK inhibitors compared to those individuals afflicted with patchy alopecia areata. Our analysis suggests that outcome indicators such as SALT_50_ obtained by observation studies may be higher than RCTs, given that patients’ disease severity in case reports or series may be severe, and display a marked inflammatory response to JAKi and more overt symptoms. Thirdly, observer bias may limit our results due to the lack of blinding in observational studies’ treatment and outcome assessment process. Lastly, the studies we included in our analysis did not provide sufficient data on the long-term efficacy, safety, and disease recurrence of JAKi drugs; hence, future large-scale, long-term RCTs are imperative to validate the efficacy and safety of these drugs.

## Conclusion

In this systematic evaluation and meta-analysis, we demonstrated the effectiveness of oral JAK inhibitors in patients with alopecia areata and that adverse effects were within manageable limits. However, we regard additional high-quality randomized controlled trials involving larger samples as crucial steps towards the identification of the ideal drug types and doses that would optimize therapeutic efficiency while limiting potential harm posed by JAK suppression.

## Author contributions

M-QM gathered the information and wrote the original draft. JJ is responsible for funding acquisition and modification. All authors are contributed to the article, participated in resources, wrote the review and edited. All authors contributed to the article and approved the submitted version.
