# Supplementary material for: The evaluation of JAK inhibitors on effect and safety in alopecia areata: a systematic review and meta-analysis of 2018 patients
Source: Front Immunol. 2023 Jun 2;14:1195858. doi: 10.3389/fimmu.2023.1195858 (PMC10272608; doi:10.3389/fimmu.2023.1195858)

**Mendeley Supplemental Data 1.** Articles included in the review.

**Mendeley Supplemental Data 2.** A structured summary if this meta-analysis.

**Mendeley Supplemental Method 1.** Data source and search strategy.

**Mendeley Supplemental Method 2.** Study selection.

**Mendeley Supplemental Method 3.** Quality assessment.

**Mendeley Supplemental Method 4.** Statistic analysis.

**Mendeley Supplemental Method 5.** Dealing with duplication

**Mendeley Supplemental Method 6.** The calculation of the Severity in Alopecia Tool (SALT).

**Mendeley Supplemental Table 1.** PRISMA statement for reporting of systematic reviews and meta-analyses.

**Mendeley Supplemental Table 2.** Study search list.

**Mendeley Supplemental Table 3.** Study characteristics and treatment protocols of included studies in this systematic review and meta-analysis.

**Mendeley Supplemental Table 4** Subgroup analysis of changes in SALT score based on drug, tofacitinib dosage, treatment duration, sex and age.

**Mendeley Supplemental Table 5** Subgroup analysis in SALT_5_.

**Mendeley Supplemental Table 6** Subgroup analysis in SALT_50_.

**Mendeley Supplemental Table 7** Subgroup analysis in SALT_90_.

**Mendeley Supplemental Table 8** Total adverse events.

**Mendeley Supplemental Table 9** Subgroup analysis of total infection based on drug, tofacitinib dosage, treatment duration, sex and age.

**Mendeley Supplemental Table 10** Subgroup analysis of laboratory abnormalities.

**Mendeley Supplemental Table 11** Subgroup analysis of neurological symptoms.

**Mendeley Supplemental Table 12** Subgroup analysis of gastrointestinal symptoms..

**Mendeley Supplemental Table 13** Subgroup analysis of cutaneous symptoms.

**Mendeley Supplemental Figure 1.** Flowchart of meta-analysis.

**Mendeley Supplemental Figure 2.** Risk of bias assessment and summary of all included randomized controlled trials.

**Mendeley Supplemental Figure 3.** Quality assessment of non-randomized controlled trials.

**Mendeley Supplemental Figure 4.** Forest plot of the hair growth rate of JAKi treatment in randomized clinical trials.

**Mendeley Supplemental Figure 5.** Forest plot of SALT_5_(A), SALT_50_(B), SALT_90_(C) rate of JAK inhibitors treatment in patients with AA in observational studies.

**Mendeley Supplemental Figure 6.** Pooled relative risk of total infection, stratified by drugs.

**Mendeley Supplemental Figure 7.** Pooled relative risk of laboratory abnormalities, stratified by drugs.

**Mendeley Supplemental Figure 8.** Pooled relative risk of neurological symptoms, stratified by drugs.

**Mendeley Supplemental Figure 9.** Pooled relative risk of gastrointestinal symptoms or weight gain, stratified by drugs.

**Mendeley Supplemental Figure 10.** Pooled relative risk of cutaneous symptoms, stratified by drugs.

**Mendeley Supplemental Figure 11.** Begg’s test of SALT_50_ in randomized controlled trials.

**Mendeley Supplemental Figure 12.** Begg’s test of SALT_50_ in non-randomized controlled trials.

**Mendeley Supplemental Figure 13.** Funnel plot of SALT_50_ in non-randomized controlled trials.

**Mendeley Supplemental Data 1.** Articles included in the review.

**Random Controlled Trails:**

[1] King B, Ohyama M, Kwon O, Zlotogorski A, Ko J, Mesinkovska NA, et al. Two Phase 3 Trials of Baricitinib for Alopecia Areata. N Engl J Med. 2022;386(18):1687-99. Epub 20220326.

[2] King B, Ko J, Forman S, Ohyama M, Mesinkovska N, Yu G, et al. Efficacy and safety of the oral Janus kinase inhibitor baricitinib in the treatment of adults with alopecia areata: Phase 2 results from a randomized controlled study. Journal of the American Academy of Dermatology. 2021;85(4):847‐53.

[3] Guttman-Yassky E, Pavel AB, Diaz A, Zhang N, Del Duca E, Estrada Y, et al. Ritlecitinib and brepocitinib demonstrate significant improvement in scalp alopecia areata biomarkers. Journal of allergy and clinical immunology. 2021.

[4] King B, Guttman-Yassky E, Peeva E, Banerjee A, Sinclair R, Pavel AB, et al. A phase 2a randomized, placebo-controlled study to evaluate the efficacy and safety of the oral Janus kinase inhibitors ritlecitinib and brepocitinib in alopecia areata: 24-week results. Journal of the American Academy of Dermatology. 2021;85(2):379-87.

[5] King B, Kwon O, Mesinkovska N, Ko J, Dutronc Y, Wu W, et al. LB785 Efficacy and safety of baricitinib in adults with Alopecia Areata: phase 3 results from a randomized controlled trial (BRAVE-AA1). Journal of investigative dermatology. 2021;141(9):B18‐.

[6] Gold M, Grande K, Moran S, Shanler SD, Burt D. 16384 ATI-501, a novel Janus kinase 1/3 inhibitor, demonstrates hair growth in patients with alopecia areata: results of a phase 2, randomized, double-blind, placebo-controlled trial. Journal of the American Academy of Dermatology. 2020;83(6):AB62‐.

**Observation Studies:**

[1] Mikhaylov D, Pavel A, Yao C, Kimmel G, Nia J, Hashim P, et al. A randomized placebo-controlled single-center pilot study of the safety and efficacy of apremilast in subjects with moderate-to-severe alopecia areata. Archives of Dermatological Research. 2019;311(1):29-36.

[2] AlMarzoug A, AlOrainy M, AlTawil L, AlHayaza G, AlAnazi R, AlIssa A, et al. Alopecia areata and tofacitinib: a prospective multicenter study from a Saudi population. International Journal of Dermatology. 2021.

[3] Wambier CG, Craiglow BG, King BA. Combination tofacitinib and oral minoxidil treatment for severe alopecia areata. Journal of the American Academy of Dermatology. 2021;85(3):743-5.

[4] Oba MC, Askin O, Balci Ekmekci O, Serdaroglu S. Correlation between serum granulysin level and clinical activity in patients with alopecia areata before and after tofacitinib therapy. Journal of Cosmetic Dermatology. 2021;20(3):971-5.

[5] Dincer Rota D, Emeksiz MAC, Erdogan FG, Yildirim D. Experience with oral tofacitinib in severe alopecia areata with different clinical responses. Journal of Cosmetic Dermatology. 2021;20(9):3026-33.

[6] Lai VWY, Bokhari L, Sinclair R. Sublingual tofacitinib for alopecia areata: a roll-over pilot clinical trial and analysis of pharmacokinetics. International Journal of Dermatology. 2021;60(9):1135-9.

[7] Jerjen R, Meah N, Trindade de Carvalho L, Wall D, Eisman S, Sinclair R. Treatment of alopecia areata in pre-adolescent children with oral tofacitinib: A retrospective study. Pediatric Dermatology. 2021;38(1):103-8.

[8] Kibbie J, Kines K, Norris D, Dunnick CA. Oral tofacitinib for the treatment of alopecia areata in pediatric patients. Pediatric Dermatology. 2022;39(1):31-4.

[9] Akdogan N, Ersoy-Evans S, Doğan S, Atakan N. Experience with oral tofacitinib in two adolescents and seven adults with alopecia areata. Dermatologic Therapy. 2019;32(6).

[10] Shin JW, Huh CH, Kim MW, Lee JS, Kwon O, Cho S, et al. Comparison of the Treatment Outcome of Oral Tofacitinib with Other Conventional Therapies in Refractory Alopecia Totalis and Universalis: A Retrospective Study. Acta Derm Venereol. 2019;99(1):41-6.

[11] Almutairi N, Nour TM, Hussain NH. Janus Kinase Inhibitors for the Treatment of Severe Alopecia Areata: An Open-Label Comparative Study. Dermatology. 2019;235(2):130-6.

[12] Jabbari A, Sansaricq F, Cerise J, Chen JC, Bitterman A, Ulerio G, et al. An Open-Label Pilot Study to Evaluate the Efficacy of Tofacitinib in Moderate to Severe Patch-Type Alopecia Areata, Totalis, and Universalis. Journal of Investigative Dermatology. 2018;138(7):1539-45.

[13] Liu LY, King BA. Ruxolitinib for the treatment of severe alopecia areata. Journal of the American Academy of Dermatology. 2019;80(2):566-8.

[14] Craiglow BG, King BA. Tofacitinib for the treatment of alopecia areata in preadolescent children. Journal of the American Academy of Dermatology. 2019;80(2):568-70.

[15] Dai YX, Chen CC. Tofacitinib therapy for children with severe alopecia areata. Journal of the American Academy of Dermatology. 2019;80(4):1164-6.

[16] Craiglow BG, Liu LY, King BA. Tofacitinib for the treatment of alopecia areata and variants in adolescents. Journal of the American Academy of Dermatology. 2017;76(1):29-32.

[17] Liu LY, Craiglow BG, Dai F, King BA. Tofacitinib for the treatment of severe alopecia areata and variants: A study of 90 patients. Journal of the American Academy of Dermatology. 2017;76(1):22-8.

[18] Park HS, Kim MW, Lee JS, Yoon HS, Huh CH, Kwon O, et al. Oral tofacitinib monotherapy in Korean patients with refractory moderate-to-severe alopecia areata: A case series. Journal of the American Academy of Dermatology. 2017;77(5):978-80.

[19] Liu LY, Craiglow BG, King BA. Successful treatment of moderate-to-severe alopecia areata improves health-related quality of life. Journal of the American Academy of Dermatology. 2018;78(3):597-9.e2.

[20] Jabbari A, Cerise J, Chen JC, Sansaricq F, Clynes R, Christiano AM, et al. 331 An open label clinical trial of the JAK inhibitor tofacitinib for alopecia areata. Journal of Investigative Dermatology. 2017;137(5, Supplement 1):S56.

[21] Ibrahim O, Bayart CB, Hogan S, Piliang M, Bergfeld WF. Treatment of alopecia areata with tofacitinib. JAMA Dermatology. 2017;153(6):600-2.

[22] Kennedy Crispin M, Ko JM, Craiglow BG, Li S, Shankar G, Urban JR, et al. Safety and efficacy of the JAK inhibitor tofacitinib citrate in patients with alopecia areata. JCI insight. 2016;1(15):e89776.

[23] Mackay-Wiggan J, Jabbari A, Nguyen N, Cerise JE, Clark C, Ulerio G, et al. Oral ruxolitinib induces hair regrowth in patients with moderate-to-severe alopecia areata. JCI insight. 2016;1(15):e89790.

[24] Castelo-Soccio L. Experience with oral tofacitinib in 8 adolescent patients with alopecia universalis. J Am Acad Dermatol. 2017;76(4):754-5.

[25] Cheng MW, Kehl A, Worswick S, Goh C. Successful Treatment of Severe Alopecia Areata With Oral or Topical Tofacitinib. J Drugs Dermatol. 2018;17(7):800-3.

[26] Shivanna CB, Shenoy C, Priya RA. Tofacitinib (Selective Janus Kinase Inhibitor 1 and 3): A Promising Therapy for the Treatment of Alopecia Areata: A Case Report of Six Patients. Int J Trichology. 2018;10(3):103-7.

**Mendeley Supplemental Data 2.** A structured summary if this meta-analysis.

*Background*: JAK inhibitors treat various autoimmune diseases, but an updated systematic review in treating alopecia areata is currently lacking.

*Objective*: Evaluate the specific efficacy and safety of JAK inhibitors in alopecia areata by systematic review and meta-analysis.

*Methods*: Eligible studies in PubMed, Embase, Web of science, and Clinical Trials up to May 30, 2022, were searched. We enrolled in randomized controlled trials and observational studies of applying JAK inhibitors in alopecia areata.

*Results*: 6 randomized controlled trials with 1455 patients exhibited SALT_50_ (odd ratio [OR], 5.08; 95% confidence interval [CI], 3.49-7.38), SALT_90_ (OR, 7.40; 95% CI, 4.34-12.67) and Change in SALT score (weighted mean difference [WSD], 5.55; 95% CI, 2.60-8.50) compared to the placebo. The proportion of 26 observational studies with 563 patients of SALT_5_ was 0.71(95% CI, 0.65-0.78), SALT_50_ was 0.54(95% CI 0.46-0.63), SALT_90_ was 0.33(95% CI, 0.24-0.42), and SALT score (WSD, -2.18; 95% CI, -3.12 to -1.23) compared with baseline. Any adverse effects occurred in 921 of 1508 patients; a total of 30 patients discontinued the trial owing to adverse reactions.

*Limitations*: Relatively few randomized controlled trials met the inclusion criteria and insufficiency of eligible data.

*Conclusion*: JAK inhibitors are effective in alopecia areata, although associated with an increased risks.

**Mendeley Supplemental Method 1.** Data source and search strategy

This study used PubMed, Embase, Web of science, and Clinical Trials databases for searching dates up to May 30, 2022. The keywords selected were "Alopecia areata" and "JAK inhibitor" or "ruxolitinib or tofacitinib or baricitinib". Subsequently, we reviewed the reference lists of all retrieved articles to identify potentially relevant studies further. The assessment was performed using inclusion and exclusion criteria.

**Mendeley Supplemental Method 2.** Study selection

Eligibility criteria consisted of (I) Studied patients with AA were pathological examination confirmed. (II) Change in SALT was used as an indicator. (III) Inclusion of case reports, case series, cohort studies, or clinical trials of patients with AA and JAKi. The titles and abstracts of included articles were screened independently by two authors (Mei-qi Mao and Jing Jing). A subsequent full-text review was performed when the abstracts provided insufficient information.

Exclusive criteria included (I) Review, meta-analysis, systematic evaluation, meeting abstracts, and case reports (less than 3 cases). (II) Clinical trials of non-oral JAKi. (III) Clinical trials to detect the effect of JAKi application on eyebrows and eyelashes. (IV) Studies were not written in Chinese or English. (V) Studies had duplicated data or repeat analyses. (VI) Studies with insufficient data

**Mendeley Supplemental Method 3.** Quality assessment.

Two authors (Mei-qi Mao and Yu-xin) performed the quality assessment independently. Any disagreements were discussed with the third author (Jing Jing) and resolved by consensus. The RCTs were assessed using the Cochrane Collaboration Risk-of-bias Instrument. OSs quality assessment were based on the Joanna Briggs Institute Critical Appraisal tools.

**Mendeley Supplemental Method 4.** Statistic analysis.

For continuous data, such as the SALT score improvement pre- and post-treatment, we estimated the weighted mean difference (WMD) with a 95% confidence interval (CI). For dichotomous variables such as rate of SALT_5_, SALT_50_, SALT_90_ and adverse events, we used pooled odds ratios (ORs) with 95% CIs. Random effects analysis was applied using Dorsmanin and Laird method^[7]^. Subgroup analysis dealt with heterogeneity, and *P*<0.05 was considered statistically significant. Publication bias was assessed using funnel plots and Begg’s plots, and *P*<0.1 if the funnel plots were asymmetric. Outcome data were subjected to meta-analysis using Stata17 (StataCorp LLC, Texas, USA).

**Mendeley Supplemental Method 5.** Dealing with duplication.

When duplication was observed, we included studies with more comprehensive information. Some studies presented the combined results of two or three clinical trials. If the combined and separate studies provided the same outcomes, we used data from the individual trials.

**Mendeley Supplemental Method 6.** The calculation of the Severity in Alopecia Tool (SALT).

The SALT score was calculated by measuring the percentage of hair loss in four areas of the scalp, including the top (40%), left side (18%), right side (18%), and back (24%) areas, higher SALT scores indicate more severe hair loss.

SALT improvement rate = (SALT score at baseline - SALT score at endpoint)/SALT score at baseline * 100%. The SALT improvement rate < 5% indicates that patients have no clinical response; the improvement rate ≥ 5% indicates a clinical response; the improvement rate ≥ 50% is good relief; the improvement rate ≥ 90% indicates complete relief of alopecia areata. We counted the number of patients with a clinical response before and after treatment, essential to measure the drug's efficacy.

**Mendeley Supplemental Table 1.** PRISMA extension statement for reporting of systematic reviews incorporating meta-analyses.

| **Section/Topic** | **Item #** | **Checklist Item** | **Reported on Page #** |
| --- | --- | --- | --- |
| **TITLE** |  |  |  |
| Title | 1 | Identify the report as a systematic review*.* | P1 |
| **ABSTRACT** |  |  |  |
| Structured summary | 2 | Provide a structured summary including, as applicable: | eData2 |
| **INTRODUCTION** |  |  |  |
| Rationale | 3 | Describe the rationale for the review in the context of what is already known*.* | P2 |
| Objectives | 4 | Provide an explicit statement of questions being addressed, with reference to participants, interventions, comparisons, outcomes, and study design (PICOS). | P2 |
| **METHODS** |  |  |  |
| Protocol and registration | 5 | Indicate whether a review protocol exists and if and where it can be accessed (e.g., Web address); and, if available, provide registration information, including registration number. | P2 |
| Eligibility criteria | 6 | Specify study characteristics (e.g., PICOS, length of follow-up) and report characteristics (e.g., years considered, language, publication status) used as criteria for eligibility, giving rationale. | eTable3 |
| Information sources | 7 | Describe all information sources (e.g., databases with dates of coverage, contact with study authors to identify additional studies) in the search and date last searched. | eTable2 |
| Search | 8 | Present full electronic search strategy for at least one database, including any limits used, such that it could be repeated. | eTable2 |
| Study selection | 9 | State the process for selecting studies (i.e., screening, eligibility, included in systematic review, and, if applicable, included in the meta-analysis). | eMethod2 |
| Data collection process | 10 | Describe method of data extraction from reports (e.g., piloted forms, independently, in duplicate) and any processes for obtaining and confirming data from investigators. | eMethod2 |
| Data items | 11 | List and define all variables for which data were sought (e.g., PICOS, funding sources) and any assumptions and simplifications made. | eMethod4 |
| **RESULTS†** |  |  |  |
| Study selection | 12 | Give numbers of studies screened, assessed for eligibility, and included in the review, with reasons for exclusions at each stage, ideally with a flow diagram. | eFigure1 |
| Study characteristics | 13 | For each study, present characteristics for which data were extracted (e.g., study size, PICOS, follow-up period) and provide the citations. | eTable3 |
| Risk of bias within studies | 14 | Present data on risk of bias of each study and, if available, any outcome level assessment. | eFigure2-3 |
| Results of individual studies | 15 | For all outcomes considered (benefits or harms), present, for each study: 1) simple summary data for each intervention group, and 2) effect estimates and confidence intervals. | eTable8 |
| Synthesis of results | 16 | Present results of each meta-analysis done, including confidence/credible intervals. If additional summary measures were explored (such as treatment rankings), these should also be presented. | Figure1-2. eFigure4-7, 6-10 |
| Risk of bias across studies | 17 | Present results of any assessment of risk of bias across studies for the evidence base being studied. | eFigure13-15 |
| Results of additional analyses | 18 | Give results of additional analyses, if done (e.g., sensitivity or subgroup analyses, meta-regression analyses*,* and so forth). | eTable4-13 |
| **DISCUSSION** |  |  |  |
| Summary of evidence | 19 | Summarize the main findings, including the strength of evidence for each main outcome; consider their relevance to key groups (e.g., healthcare providers, users, and policy-makers). | P2-3 |
| Limitations | 20 | Discuss limitations at study and outcome level (e.g., risk of bias), and at review level (e.g., incomplete retrieval of identified research, reporting bias). | P3,eData2 |
| Conclusions | 21 | Provide a general interpretation of the results in the context of other evidence, and implications for future research. | P3 |
| **FUNDING** |  |  |  |
| Funding | 22 | Describe sources of funding for the systematic review and other support (e.g., supply of data); role for the systematic review. | P1 |
| PICOS = population, intervention, comparators, outcomes, study design.  * Text in italics indicate S wording specific to reporting of network meta-analyses that has been added to guidance from the PRISMA statement.  † Authors may wish to plan for use of appendices to present all relevant information in full detail for items in this section. | | | |

**Mendeley Supplemental Table 2.** Study search list.

| Database | Search Strategy |
| --- | --- |
| Embase | ("alopecia areata"[Title/Abstract] AND ("tyrosine kinase inhibitor"[Title/Abstract] OR "tyrosine kinase inhibitors"[Title/Abstract] OR "tyrosine kinase inhibitor"[Title/Abstract] OR "janus kinase inhibitor"[Title/Abstract] OR "jak inhibitor"[Title/Abstract] OR "tofacitinib"[Title/Abstract] OR "baricitinib"[Title/Abstract] OR "ruxolitinib"[Title/Abstract] OR "oclacitinib"[Title/Abstract])) AND (clinicaltrial[Filter]) |
| Web of science | (TS=(’alopecia areata’)) AND TS=(‘tyrosine kinase inhibitor’ OR ‘tyrosine kinase inhibitors’ OR ‘tyrosine-kinase inhibitor’ OR ‘Janus kinase inhibitor’ OR ‘JAK inhibitor’ OR tofacitinib OR baricitinib OR ruxolitinib OR oclacitinib) and Clinical Trial |
| Clinical Trails | ‘alopecia areata’ \| ‘tyrosine kinase inhibitor’ OR ‘tyrosine kinase inhibitors’ OR ‘tyrosine-kinase inhibitor’ OR ‘Janus kinase inhibitor’ OR ‘JAK inhibitor’ OR tofacitinib OR baricitinib OR ruxolitinib OR oclacitinib |
| Pubmed | ("alopecia areata"[Title/Abstract] AND ("tyrosine kinase inhibitor"[Title/Abstract] OR "tyrosine kinase inhibitors"[Title/Abstract] OR "tyrosine kinase inhibitor"[Title/Abstract] OR "janus kinase inhibitor"[Title/Abstract] OR "jak inhibitor"[Title/Abstract] OR "tofacitinib"[Title/Abstract] OR "baricitinib"[Title/Abstract] OR "ruxolitinib"[Title/Abstract] OR "oclacitinib"[Title/Abstract])) AND (clinicaltrial[Filter]) |

*Studies involved from database creation to May 30, 2022.

**Mendeley Supplemental Table 3.** Study characteristics and treatment protocols of included studies in this systematic review and meta-analysis.

| Study reference | Patient characteristic | | | | | Treatment regiment | | | Study type |
| --- | --- | --- | --- | --- | --- | --- | --- | --- | --- |
|  | n | Males | Females | AA | AU/AT | Oral JAK inhibitor | Dosage | Duration of treatment |  |
| King, B. 2021(BRAVE-AA1) | 654 | 271 | 383 | 370 | 284 | Baricitinib | 2mg, 4mg QD | 36 weeks | RCT III (BRAVE-AA1) |
| King, B. 2022(BRAVE-AA2) | 546 | 201 | 345 | 299 | 247 | Baricitinib | 2mg, 4mg QD | 36 weeks | RCT III (BRAVE-AA2) |
| Guttman-Yassky, E. 2021 | 46 | 13 | 33 | 13 | 33 | Ritlecitinib, Brepocitinib | not mentioned | 6 months | RCT II |
| King, B. 2021 | 142 | 44 | 98 | 80 | 62 | Ritlecitinib, Brepocitinib | Ritlecitinib 200mgQD, Brepocitinib 60mgQD | 24 weeks | RCT II |
| Gold, M. 2020 | 87 | 31 | 56 | 40 | 45 | ATI-501 | 400-800mg BID | 6 months | RCT |
| King, B. 2021 | 110 | 28 | 82 | NR | NR | Baricitinib | 2mg, 4mg QD | 36 weeks | RCT |
| Mikhaylov, D., 2020 | 9 | 3 | 6 | 9 | 0 | Tofacitinib | 5-15mg BID | 5-42 months | OS |
| AlMarzoug, A., 2021 | 65 | 28 | 37 | 17 | 48 | Tofacitinib | 5mg BID | 6 months | OS |
| Wambier, C. G, 2021 | 12 | 5 | 7 | NR | NR | Tofacitinib | 5-10mg BID | 4-6 months | OS |
| Oba, M. C., 2020 | 38 | 22 | 16 | 12 | 26 | Tofacitinib | 5mg BID | 6 months | OS |
| Dincer Rota, 2021 | 13 | 5 | 8 | 3 | 10 | Tofacitinib | 10mg BID | 3-15 months | OS |
| Lai, V. W. Y., 2021 | 18 | 4 | 14 | 5 | 13 | Tofacitinib | 5mg BID | 3 months | OS |
| Jerjen, R., 2021 | 11 | 5 | 6 | 5 | 6 | Tofacitinib | 2.5-7.5mg QD | 7-38 months | OS |
| Kibbie, J., 2022 | 11 | 4 | 7 | 4 | 7 | Tofacitinib | 5-10mg BID | 5-39 months | OS |
| Akdogan, N., 2019 | 9 | 8 | 1 | 4 | 5 | Tofacitinib | 10mg QD | 6 months | OS |
| Shin, J. W., 2019 | 18 | 7 | 11 | 0 | 18 | Tofacitinib | 5mg BID | 6 months | OS |
| Almutairi, N., 2018 | 75 | 43 | 32 | 33 | 42 | Tofacitinib, Ruxolitinib | Tofacitinib 5mg BID, Ruxolitinib 20mg BID | 6 months | OS |
| Jabbari, A., 2018 | 11 | 4 | 7 | 6 | 5 | Tofacitinib | 5-10mg BID | 6-18 months | OS |
| Liu, L. Y., 2019 | 8 | 4 | 4 | 2 | 6 | Ruxolitinib | 10-25mg BID | 5-31 months | OS |
| Craiglow, B. G, 2019 | 4 | 1 | 3 | 0 | 4 | Tofacitinib | 5mg BID | 6-15 months | OS |
| Dai, Y. X., 2019 | 3 | 2 | 1 | 0 | 3 | Tofacitinib | 2.5mg BID | 6-21 months | OS |
| Craiglow, B. G., 2017 | 13 | 3 | 10 | 6 | 7 | Tofacitinib | 5mg BID | 2-16 months | OS |
| Liu, L. Y., 2017 | 65 | 33 | 32 | 13 | 78 | Tofacitinib | 5mg BID | 4-18 months | OS |
| Park, H. S., 2017 | 22 | 16 | 6 | 12 | 21 | Tofacitinib | 10mg BID | 4-17 months | OS |
| Liu, L. Y., 2018 | 30 | 16 | 14 | NR | NR | Tofacitinib | Not mention | 30 months | OS |
| Jabbari, A., 2017 | 12 | NR | NR | NR | NR | Tofacitinib | 5-10mg BID | 6 months | OS |
| Ibrahim, O., 2017 | 13 | 1 | 12 | 4 | 9 | Tofacitinib | 10-20mg BID | 0.5-13 months | OS |
| Kennedy Crispin, M., 2016 | 66 | 35 | 31 | 14 | 52 | Tofacitinib | 5mg BID | 3 months | OS |
| Mackay-Wiggan, J., 2016 | 12 | 5 | 7 | NR | NR | Ruxolitinib | 20mg BID | 3-6 months | OS |
| Castelo-Soccio, L., 2017 | 8 | NR | NR | 0 | 8 | Tofacitinib | 5mg BID | 5-18 months | OS |
| Cheng, M. W., 2018 | 11 | 3 | 8 | 0 | 11 | Tofacitinib | 5mg QD-11 mg BID | 4.5-27 months | OS |
| Shivanna, C. B., 2018 | 6 | 3 | 3 | 0 | 6 | Tofacitinib | 5-10mg BID | 3-6 months | OS |
| AA: Alopecia areata; AT: Alopecia totalis; AU: Alopecia universalis; JAK: Janus kinase; NR: Not reported; OS, observational study;RCT, randomized clinical trial. | | | | | | | | | |

**Mendeley Supplemental Table 4** Subgroup analysis of changes in SALT score based on drug, tofacitinib dosage, treatment duration, sex and age.

| Variable | number of Studies | number of Participants | WMD(95% CI) | Heterogeneity | | *P* Value |
| --- | --- | --- | --- | --- | --- | --- |
|  |  |  |  | *P* | I^2^, % |  |
| **Randomized controlled trial** | | | | | | |
| Drugs | | | | | | |
| Baricitinib | 3 | 933 | 9.54(6.48,12.59) | 0.000 | 98.6 | 0.000^***^ |
| Ritlecitinib | 1 | 18 | 0.77(0.01,1.53) | / | / |  |
| Brepocitinib | 1 | 16 | 1.26(0.44,2.09) | / | / |  |
| ATI-501 | 1 | 43 | 0.64(0.28,0.99) | / | / |  |
| **Observation studies** | | | | | | |
| Drugs | | | | | | |
| Tofacitinib | 11 | 147 | -1.99(-2.94,-1.03) | 0.000 | 90.2 | 0.488 |
| Ruxolitinib | 2 | 50 | -3.13(-6.20,-0.05) | 0.000 | 95.7 |  |
| Oral dose of Tofacitinib | | | | | | |
| >10 mg daily | 9 | 102 | -1.49(-2.07,-0.90) | 0.001 | 68.6 | 0.453 |
| <=10 mg daily | 2 | 45 | -3.67(-9.35,2.02) | 0.000 | 98.1 |  |
| Treatment duration | | | | | | |
| >6 months | 7 | 140 | -2.28(-3.70,-0.86) | 0.000 | 65.6 | 0.000^***^ |
| <=6 months | 5 | 57 | -1.89(-2.88,-0.90) | 0.002 | 76.5 |  |
| Sex ratio (Males/Females) | | | | | | |
| sex ratio<=1 | 8 | 103 | -1.50(-2.07,-0.93) | 0.002 | 68.6 | 0.171 |
| sex ratio>1 | 4 | 94 | -3.23(-5.63,-0.82) | 0.000 | 99.5 |  |
| Age | | | | | | |
| Adult | 12 | 192 | -2.32(-3.33,-1.31) | 0.000 | 91.9 | 0.007^*^ |
| Pediatrics/adolescents | 1 | 13 | -0.57(-1.35,0.22) | / | / |  |
| **P*<0.05, ****P*<0.001. | | | | | | |

**Mendeley Supplemental Table 5** Subgroup analysis in SALT_5_.

| Variable | number of Studies | number of Participants | | Event Rate(95% CI) | Heterogeneity | | *P* Value |
| --- | --- | --- | --- | --- | --- | --- | --- |
|  |  | SALT_5_ | Total |  | *P* | I^2^, % |  |
| **Observation studies** | | | | | | | |
| Oral dose of Tofacitinib | | | | | | | |
| <=10 mg daily | 6 | 193 | 283 | 0.69(0.63,0.74) | 0.507 | 0.0 | 0.456 |
| >10 mg daily | 10 | 82 | 116 | 0.73(0.62,0.85) | 0.012 | 57.2 |  |
| Treatment duration | | | | | | | |
| >6 months | 13 | 217 | 304 | 0.74(0.68,0.80) | 0.138 | 30.7 | 0.171 |
| <=6 months | 3 | 58 | 95 | 0.56(0.19,0.92) | 0.055 | 65.5 |  |
| Sex ratio (Males/Females) | | | | | | | |
| sex ratio<=1 | 11 | 126 | 176 | 0.75(0.66,0.83) | 0.044 | 46.6 | 0.169 |
| sex ratio>1 | 5 | 149 | 223 | 0.49(0.37,0.60) | 0.180 | 36.2 |  |
| Age | | | | | | | |
| Adult | 14 | 258 | 377 | 0.70(0.63,0.77) | 0.011 | 52.4 | 0.429 |
| Pediatrics/adolescents | 2 | 17 | 22 | 0.78(0.61,0.95) | 0.609 | 0 |  |

**Mendeley Supplemental Table 6** Subgroup analysis in SALT_50_.

| Variable | number of Studies | number of Participants | | Event Rate(95% CI) | Heterogeneity | | *P* Value |
| --- | --- | --- | --- | --- | --- | --- | --- |
|  |  | SALT_50_ | Total |  | *P* | I^2^, % |  |
| **Randomized controlled trial** | | | | | | | |
| Drugs | | | | | | | |
| Baricitinib | 3 | 185 | 933 | 0.20(0.14,0.26) | 0.000 | 79.2 | 0.174 |
| Ritlecitinib | 1 | 5 | 18 | 0.28(0.07,0.48) | / | / |  |
| Brepocitinib | 1 | 6 | 16 | 0.38(0.14,0.61) | / | / |  |
| ATI-501 | 2 | 41 | 132 | 0.30(0.22,0.38) | 0.596 | 0 |  |
| **Observation studies** | | | | | | | |
| Drugs | | | | | | | |
| Tofacitinib | 21 | 265 | 558 | 0.52(0.43,0.61) | 0.000 | 75.9 | 0.114 |
| Ruxolitinib | 3 | 39 | 65 | 0.64(0.52,0.75) | 0.423 | 0 |  |
| Oral dose of Tofacitinib | | | | | | | |
| <=10 mg daily | 7 | 171 | 340 | 0.51(0.42,0.59) | 0.018 | 60.7 | 0.21 |
| >10 mg daily | 14 | 82 | 160 | 0.53(0.38,0.68) | 0.000 | 80.8 |  |
| Treatment duration | | | | | | | |
| >6 months | 19 | 264 | 509 | 0.53(0.45,0.60) | 0.000 | 65.6 | 0.878 |
| <=6 months | 4 | 28 | 53 | 0.56(0.19,0.92) | 0.000 | 91.2 |  |
| Sex ratio (Males/Females) | | | | | | | |
| sex ratio<=1 | 16 | 123 | 231 | 0.56(0.45,0.68) | 0.000 | 73.6 | 0.341 |
| sex ratio>1 | 8 | 169 | 331 | 0.49(0.37,0.60) | 0.000 | 77.9 |  |
| Age | | | | | | | |
| Adult | 20 | 268 | 517 | 0.51(0.47,0.55) | 0.000 | 77.2 | 0.08 |
| Pediatrics/adolescents | 2 | 15 | 22 | 0.69(0.49,0.88) | 0.646 | 0 |  |

**Mendeley Supplemental Table 7** Subgroup analysis in SALT_90_.

| Variable | number of Studies | number of Participants | | Event Rate(95% CI) | Heterogeneity | | *P* Value |
| --- | --- | --- | --- | --- | --- | --- | --- |
|  |  | SALT_90_ | Total |  | *P* | I^2^, % |  |
| **Observation studies** | | | | | | | |
| Drugs | | | | | | | |
| Tofacitinib | 13 | 83 | 279 | 0.33(0.33,0.43) | 0.000 | 71.9 | 0.806 |
| Ruxolitinib | 2 | 15 | 50 | 0.38(0.01,0.74) | 0.018 | 82.3 |  |
| Oral dose of Tofacitinib | | | | | | | |
| >10 mg daily | 8 | 28 | 80 | 0.38(0.18,0.58) | 0.000 | 80.6 | 0.584 |
| <=10 mg daily | 5 | 55 | 199 | 0.28(0.19,0.36) | 0.153 | 40.2 |  |
| Treatment duration | | | | | | | |
| >6 months | 11 | 87 | 292 | 0.34(0.24,0.44) | 0.000 | 69.9 | 0.701 |
| <=6 months | 3 | 11 | 37 | 0.28(0.00,0.57) | 0.006 | 80.4 |  |
| Sex ratio (Males/Females) | | | | | | | |
| sex ratio<=1 | 10 | 46 | 145 | 0.38(0.22,0.54) | 0.000 | 80.9 | 0.280 |
| sex ratio>1 | 5 | 52 | 184 | 0.28(0.20,0.35) | 0.260 | 23.1 |  |
| Age | | | | | | | |
| Adult | 12 | 92 | 315 | 0.32(0.23,0.42) | 0.000 | 74.3 | 0.469 |
| Pediatrics/adolescents | 2 | 6 | 14 | 0.43(0.17,0.68) | 0.697 | 0.0 |  |

**Mendeley Supplemental Table 8** Subgroup analysis of total adverse events based on drug, tofacitinib dosage, treatment duration, sex and age.

| **Study** | **Treatment arms** | **Any AE** | **Serious AE** | **AE-related withdrawal** | **Total infections** | **Laboratory abnormalities** | **Neurological**  **Symptoms** | **Gastrointestinal Symptoms**  **or weight gain** | **Cutaneous**  **Symptoms** | **Malignancy** |
| --- | --- | --- | --- | --- | --- | --- | --- | --- | --- | --- |
| King, 2021  (BRAVE-AA1) | Placebo | 97/189 | 3/189 | 2/189 | 25/189 | 3/189 | 9/189 | NR | 1/189 | 0/189 |
| King, 2021  (BRAVE-AA1) | Baricitinib  2mg QD | 93/183 | 4/183 | 3/183 | 23/183 | 3/183 | 8/183 | NR | 10/183 | 0/183 |
| King, 2021  (BRAVE-AA1) | Baricitinib  4mg QD | 167/280 | 6/280 | 5/280 | 49/280 | 16/280 | 14/280 | NR | 16/280 | 0/280 |
| King, 2022  (BRAVE-AA2) | Placebo | 97/154 | 3/154 | 4/154 | 20/154 | 2/154 | 10/154 | NR | 3/154 | 0/154 |
| King, 2022  (BRAVE-AA1) | Baricitinib  2mg QD | 106/155 | 4/155 | 4/155 | 26/155 | 0/155 | 12/155 | NR | 9/155 | 0/155 |
| King, 2022  (BRAVE-AA1) | Baricitinib  4mg QD | 154/233 | 8/233 | 6/233 | 41/233 | 7/233 | 21/233 | NR | 11/233 | 0/233 |
| King, 2021 | Placebo | 35/47 | 0/47 | 2/47 | 13/47 | 1/47 | 13/47 | 7/47 | 3/47 | 0/47 |
| King, 2021 | Ritlecitinib  200mg QD | 32/48 | 0/48 | 0/48 | 12/48 | 0/48 | 9/48 | 4/48 | 11/48 | 0/48 |
| King, 2021 | Brepocitinib  60mg QD | 36/47 | 2/47 | 2/47 | 24/47 | 3/47 | 7/47 | 5/47 | 7/47 | 0/47 |
| King, 2021 | Placebo | 17/28 | 0/28 | 0/28 | 5/28 | NR | 0/28 | NR | 0/28 | 0/28 |
| King, 2021 | Baricitinib  2mg QD | 19/27 | 0/27 | 0/27 | 3/27 | NR | 2/27 | NR | 2/27 | 0/27 |
| King, 2021 | Baricitinib  4mg QD | 21/27 | 0/27 | 1/27 | 6/27 | NR | 2/27 | NR | 3/27 | 0/27 |
| Wambier, 2021 | Tofacitinib  5-10mg BID | 8/12 | 0/12 | 0/12 | 0/12 | 0/12 | 0/12 | 0/12 | 8/12 | 0/12 |
| Rota, 2021 | Tofacitinib  10mg BID | 11/13 | 0/13 | 0/13 | 0/13 | 2/13 | 0/13 | 0/13 | 9/13 | 0/13 |
| Jerjen, 2021 | Tofacitinib  2.5-7.5mg QD | 9/11 | 0/11 | 0/11 | 3/11 | 9/11 | 0/11 | 0/11 | 0/11 | 0/11 |
| Kibbie, 2022 | Tofacitinib  5-10mg BID | 4/11 | 0/11 | 0/11 | 0/11 | 3/11 | 1/11 | 0/11 | 0/11 | 0/11 |
| Akdogan, 2019 | Tofacitinib  10mg QD | 3/9 | 0/9 | 0/9 | 2/9 | 1/9 | 0/9 | 0/9 | 0/9 | 0/9 |
| Shin, 2019 | Tofacitinib  5mg BID | 6/18 | 0/18 | 0/18 | 2/18 | 0/18 | 1/18 | 1/18 | 3/18 | 0/18 |
| Almutair, 2018 | Tofacitinib  5mg BID, | NR | 0/37 | 0/37 | 16/37 | 7/37 | 2/37 | 2/37 | 0/37 | 0/37 |
| Almutair, 2018 | Ruxolitinib  20mg BID | NR | 0/38 | 0/38 | 9/38 | 5/38 | 4/38 | 3/38 | 0/38 | 0/38 |
| Jabbari, 2018 | Tofacitinib  5-10mg BID | NR | 1/12 | 1/12 | 12/12 | 1/12 | 3/12 | 7/12 | 3/12 | 0/12 |
| Dai, 2018 | Tofacitinib  2.5mg BID | 3/3 | 0/3 | 0/3 | 1/3 | 0/3 | 0/3 | 2/3 | 0/3 | 0/3 |
| Craiglow, 2017 | Tofacitinib  5mg BID | NR | 0/13 | 0/13 | 4/13 | 4/13 | 3/13 | 0/13 | 0/13 | 0/13 |
| Liu, 2017 | Tofacitinib  5mg BID | NR | 0/90 | NR | 36/90 | 38/90 | 28/90 | 8/90 | 10/90 | 0/90 |
| Ibrahim, 2017 | Tofacitinib 10-20mg BID | 3/13 | 0/13 | 0/13 | 0/13 | 2/13 | 0/13 | 0/13 | 1/13 | 0/13 |
| Kennedy, 2016 | Tofacitinib  5mg BID, | NR | 0/66 | 0/66 | 17/66 | NR | 5/66 | 9/66 | 5/66 | 0/66 |
| Mackay-Wiggan, 2016 | Ruxolitinib  20mg BID | NR | 0/12 | 0/12 | 11/12 | 1/12 | 0/12 | 0/12 | 3/12 | 0/12 |
| Total infections:upper respiratory infection, urinary tract infection, tonsillitis, varicella zoster, bronchitis, opportunistic infections, nasopharyngitis, genital warts, conjunctivitis; Laboratory abnormalities: AST/ALT elevation, elevated LDL/HDL/TG/cholesterol, decreased blood cell number, increased blood creatine kinase; Neurologic symptoms:headache, fatigue, numbness, dizziness, tinnitus, neuropathic pain; Gastrointestinal symptoms:abdominal pain, nausea, diarrhea, increased bowel movement frequency, loose stools, bloating, constipation; Cutaneous symptoms: acne, skin rash and irritation, folliculitis, palmoplantar desquamation, urticaria. LDL: Low-density lipoprotein, HDL: High-density lipoprotein, TG: Triglycerides, AST: Aspartate aminotransferase, ALT: Alanine aminotransferase; NR, not reported. | | | | | | | | | | |

**Mendeley Supplemental Table 9** Subgroup analysis of total infection based on drug, tofacitinib dosage, treatment duration, sex and age.

| Variable | number of Studies | number of Participants | | Event Rate(95% CI) | Heterogeneity | | *P* Value |
| --- | --- | --- | --- | --- | --- | --- | --- |
|  |  | Event | Total |  | *P* | I^2^, % |  |
| **Infection** |  | | | | | | |
| Drugs | | | | | | | |
| Placebo | 3 | 63 | 418 | 0.15(0.10,0.20) | 0.183 | 38.2 | 0.000^***^ |
| Baricitinib | 3 | 148 | 905 | 0.16(0.14,0.18) | 0.531 | 0 |  |
| Ritlecitinib | 1 | 12 | 48 | 0.25(0.13,0.37) | / | / |  |
| Brepocitinib | 1 | 24 | 47 | 0.51(0.37,0.65) | / | / |  |
| Tofacitinib | 8 | 81 | 247 | 0.29(0.21,0.38) | 0.053 | 49.6 |  |
| Ruxolitinib | 2 | 20 | 50 | 0.58(-0.09,1.24) | 0.000 | 97.6 |  |
| Sex ratio(Males/Females) | | | | | | | |
| sex ratio<=1 | 20 | 281 | 1542 | 0.24(0.18,0.29) | 0.000 | 87.2 | 0.080 |
| sex ratio>1 | 4 | 79 | 234 | 0.33(0.24,0.41) | 0.133 | 43.2 |  |
| Age | | | | | | | |
| Adult | 21 | 352 | 1749 | 0.25(0.20,0.31) | 0.000 | 88.3 | 0.642 |
| Pediatrics/adolescents | 3 | 8 | 27 | 0.30(0.12,0.47) | 0.972 | 0.0 |  |
| Treatment duration | | | | | | | |
| >6 months | 22 | 332 | 1698 | 0.21(0.17,0.26) | 0.000 | 72.4 | 0.263 |
| <6 months | 2 | 28 | 78 | 0.58(-0.06,1.23) | 0.000 | 97.9 |  |
| **P*<0.05, ****P*<0.001. | | | | | | | |

**Mendeley Supplemental Table 10** Subgroup analysis of laboratory abnormalities.

| Variable | number of Studies | number of Participants | | Event Rate(95% CI) | Heterogeneity | | *P* Value |
| --- | --- | --- | --- | --- | --- | --- | --- |
|  |  | Event | Total |  | *P* | I^2^, % |  |
| **Laboratory abnormalities** | | | | | | | |
| Drugs | | | | | | | |
| Placebo | 3 | 6 | 390 | 0.02(0.00,0.03) | 0.929 | 0 | 0.000^***^ |
| Baricitinib | 2 | 26 | 696 | 0.03(0.01,0.05) | 0.052 | 66.2 |  |
| Brepocitinib | 1 | 3 | 47 | 0.06(-0.01,0.13) | / | / |  |
| Tofacitinib | 9 | 67 | 209 | 0.27(0.14,0.41) | 0.000 | 81.3 |  |
| Ruxolitinib | 2 | 6 | 50 | 0.12(0.03,0.20) | 0.618 | 0 |  |
| Oral dose of Tofacitinib | | | | | | | |
| <=10 mg daily | 5 | 59 | 160 | 0.36(0.16,0.57) | 0.000 | 86.5 | 0.053 |
| >10 mg daily | 4 | 8 | 49 | 0.14(0.05,0.24) | 0.679 | 0 |  |
| Sex ratio(Males/Females) | | | | | | | |
| sex ratio<=1 | 11 | 58 | 1463 | 0.05(0.03,0.08) | 0.000 | 80.1 | 0.039^*^ |
| sex ratio>1 | 3 | 50 | 165 | 0.25(0.06,0.43) | 0.000 | 87.8 |  |
| Age | | | | | | | |
| Adult | 10 | 92 | 1357 | 0.07(0.04,0.09) | 0.000 | 83.2 | 0.027^*^ |
| Pediatrics/adolescents | 3 | 16 | 35 | 0.47(0.11,0.83) | 0.002 | 84.1 |  |
| Treatment duration | | | | | | | |
| >6 months | 14 | 105 | 1603 | 0.09(0.06,0.13) | 0.000 | 88.9 | 0.803 |
| <6 months | 2 | 3 | 25 | 0.11(-0.01,0.23) | 0.582 | 0.0 |  |
| **P*<0.05, ****P*<0.001. | | | | | | | |

**Mendeley Supplemental Table 11** Subgroup analysis of neurological symptoms.

| Variable | number of Studies | number of Participants | | Event Rate(95% CI) | Heterogeneity | | *P* Value |
| --- | --- | --- | --- | --- | --- | --- | --- |
|  |  | Event | Total |  | *P* | I^2^, % |  |
| **Neurologic Symptoms** | | | | | | | |
| Drugs | | | | | | | |
| Placebo | 3 | 52 | 390 | 0.10(0.03,0.17) | 0.003 | 82.9 | 0.043^*^ |
| Baricitinib | 3 | 59 | 905 | 0.06(0.04,0.08) | 0.399 | 2.8 |  |
| Brepocitinib | 1 | 7 | 47 | 0.19(0.08,0.30) | / | / |  |
| Tofacitinib | 7 | 43 | 247 | 0.14(0.05,0.22) | 0.001 | 74.7 |  |
| Ruxolitinib | 1 | 4 | 38 | 0.11(0.01,0.20) | / | / |  |
| Oral dose of Tofacitinib | | | | | | | |
| >10 mg daily | 2 | 4 | 23 | 0.14(0.00,0.29) | 0.296 | 8.6 | 0.906 |
| <=10 mg daily | 5 | 39 | 224 | 0.13(0.03,0.24) | 0.679 | 0 |  |
| Sex ratio(Males/Females) | | | | | | | |
| sex ratio<=1 | 8 | 117 | 1444 | 0.08(0.06,0.10) | 0.011 | 51.6 | 0.319 |
| sex ratio>1 | 3 | 39 | 231 | 0.13(0.03,0.24) | 0.000 | 85.2 |  |
| Age | | | | | | | |
| Adult | 9 | 151 | 1735 | 0.09(0.07,0.12) | 0.000 | 69.7 | 0.494 |
| Pediatrics/adolescents | 2 | 4 | 24 | 0.14(0.00,0.28) | 0.336 | 0.0 |  |
| Treatment duration | | | | | | | |
| >6 months | 9 | 149 | 1685 | 0.10(0.07,0.12) | 0.000 | 68.9 | 0.577 |
| <6 months | 1 | 5 | 66 | 0.08(0.01,0.14) | / | / |  |
| **P*<0.05, ****P*<0.001. | | | | | | | |

**Mendeley Supplemental Table 12** Subgroup analysis of gastrointestinal symptoms.

| Variable | number of Studies | number of Participants | | Event Rate(95% CI) | Heterogeneity | | *P* Value |
| --- | --- | --- | --- | --- | --- | --- | --- |
|  |  | Event | Total |  | *P* | I^2^, % |  |
| **Gastrointestinal Symptoms or weight gain** | | | | | | | |
| Drugs | | | | | | | |
| Placebo | 1 | 7 | 47 | 0.15(0.05,0.25) | / | / | 0.774 |
| Ritlecitinib | 1 | 4 | 48 | 0.08(0.01,0.16) | / | / |  |
| Brepocitinib | 1 | 5 | 47 | 0.11(0.02,0.19) | / | / |  |
| Tofacitinib | 6 | 29 | 226 | 0.13(0.05,0.22) | 0.002 | 73.6 |  |
| Ruxolitinib | 1 | 3 | 38 | 0.08(-0.01,0.16) | / | / |  |
| Oral dose of Tofacitinib | | | | | | | |
| >10 mg daily | 1 | 7 | 12 | 0.58(0.30,0.86) | / | / | 0.001^***^ |
| <=10 mg daily | 5 | 22 | 214 | 0.09(0.04,0.15) | 0.134 | 43.1 |  |
| Sex ratio(Males/Females) | | | | | | | |
| sex ratio<=1 | 3 | 24 | 172 | 0.14(0.05,0.22) | 0.011 | 69.5 | 0.423 |
| sex ratio>1 | 4 | 24 | 234 | 0.09(0.04,0.14) | 0.153 | 40.2 |  |
| Age | | | | | | | |
| Adult | 5 | 45 | 385 | 0.11(0.06,0.15) | 0.033 | 54.1 | 0.519 |
| Pediatrics/adolescents | 2 | 3 | 21 | 0.30(-0.29,0.89) | 0.028 | 79.4 |  |
| Treatment duration | | | | | | | |
| >6 months | 6 | 39 | 340 | 0.11(0.06,0.16) | 0.013 | 58.6 | 0.551 |
| <6 months | 1 | 9 | 66 | 0.14(0.05,0.22) | / | / |  |
| **P*<0.05, ****P*<0.001. | | | | | | | |

**Mendeley Supplemental Table 13** Subgroup analysis of cutaneous symptoms.

| Variable | number of Studies | number of Participants | | Event Rate(95% CI) | Heterogeneity | | *P* Value |
| --- | --- | --- | --- | --- | --- | --- | --- |
|  |  | Event | Total |  | *P* | I^2^, % |  |
| **Cutaneous Symptoms** | | | | | | | |
| Drugs | | | | | | | |
| Placebo | 3 | 10 | 390 | 0.01(0.00,0.03) | 0.154 | 46.6 | 0.000^***^ |
| Baricitinib | 3 | 51 | 905 | 0.06(0.04,0.07) | 0.928 | 0 |  |
| Brepocitinib | 1 | 7 | 47 | 0.15(0.05,0.25) | / | / |  |
| Tofacitinib | 7 | 39 | 224 | 0.25(0.12,0.37) | 0.000 | 84.8 |  |
| Ruxolitinib | 1 | 3 | 12 | 0.25(0.01,0.49) | / | / |  |
| Oral dose of Tofacitinib | | | | | | | |
| >10 mg daily | 4 | 21 | 50 | 0.41(0.09,0.73) | 0.000 | 88.4 | 0.061 |
| <=10 mg daily | 3 | 18 | 174 | 0.10(0.05,0.14) | 0.540 | 0.0 |  |
| Sex ratio(Males/Females) | | | | | | | |
| sex ratio<=1 | 10 | 103 | 1470 | 0.09(0.06,0.12) | 0.000 | 85.3 | 0.831 |
| sex ratio>1 | 2 | 15 | 156 | 0.09(0.05,0.14) | 0.447 | 0.0 |  |
| Treatment duration | | | | | | | |
| >6 months | 8 | 93 | 1523 | 0.06(0.04,0.09) | 0.000 | 77.6 | 0.050* |
| <6 months | 4 | 25 | 103 | 0.41(0.07,0.75) | 0.000 | 92.1 |  |
| **P*<0.05, ****P*<0.001. | | | | | | | |

**Mendeley Supplemental Figure 1.** Flowchart of meta-analysis.


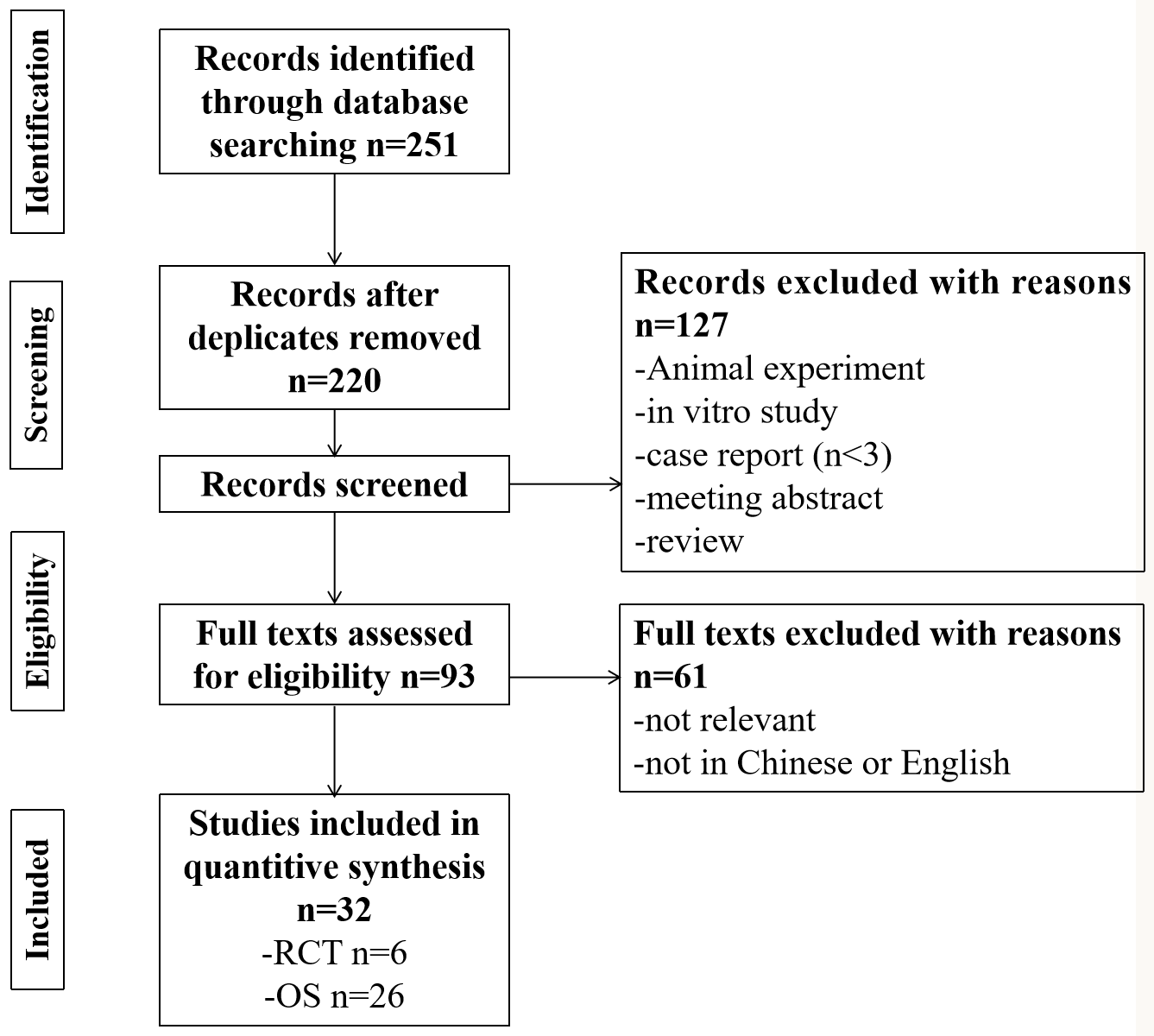


**Mendeley Supplemental Figure 2.** Risk of bias assessment and summary of all included randomized controlled trials.


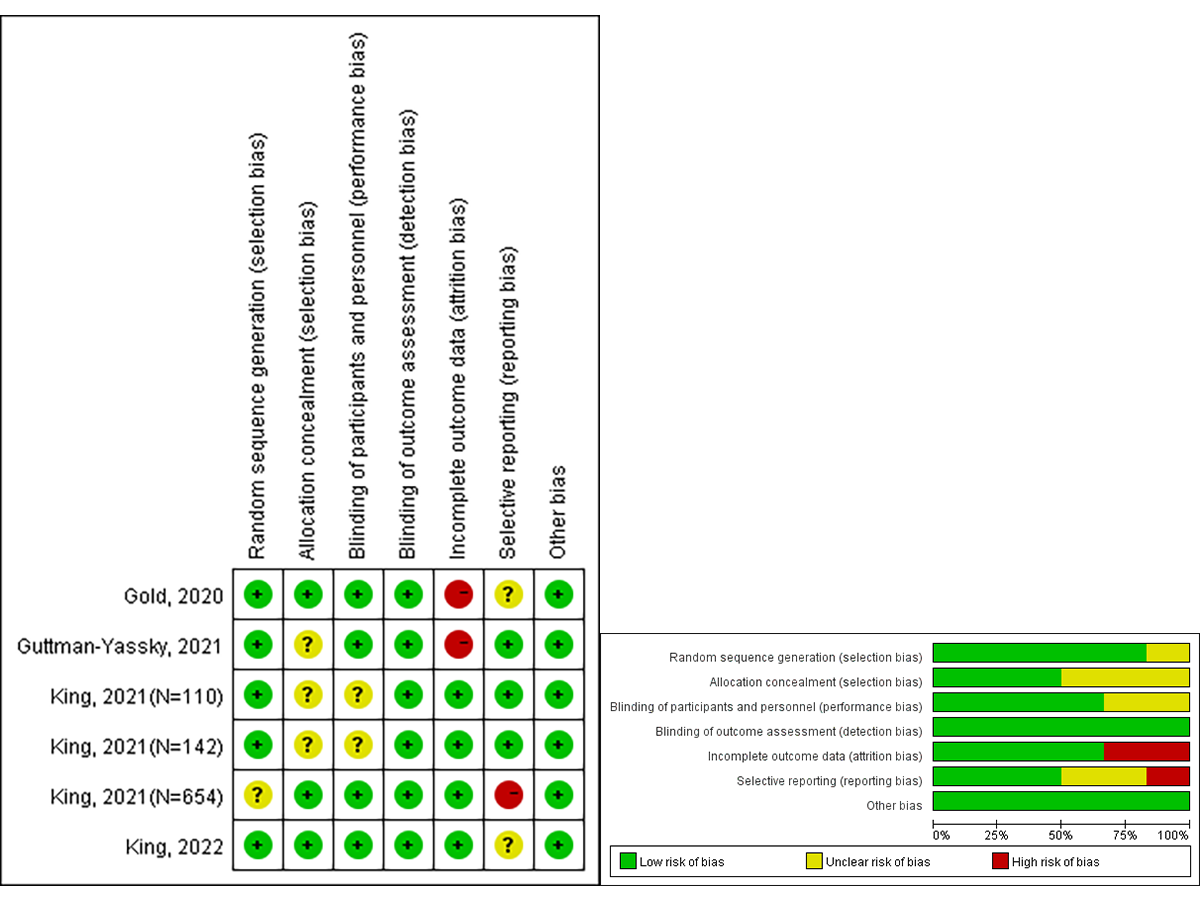


**Mendeley Supplemental Figure 3.** Quality assessment of non-randomized controlled trials.


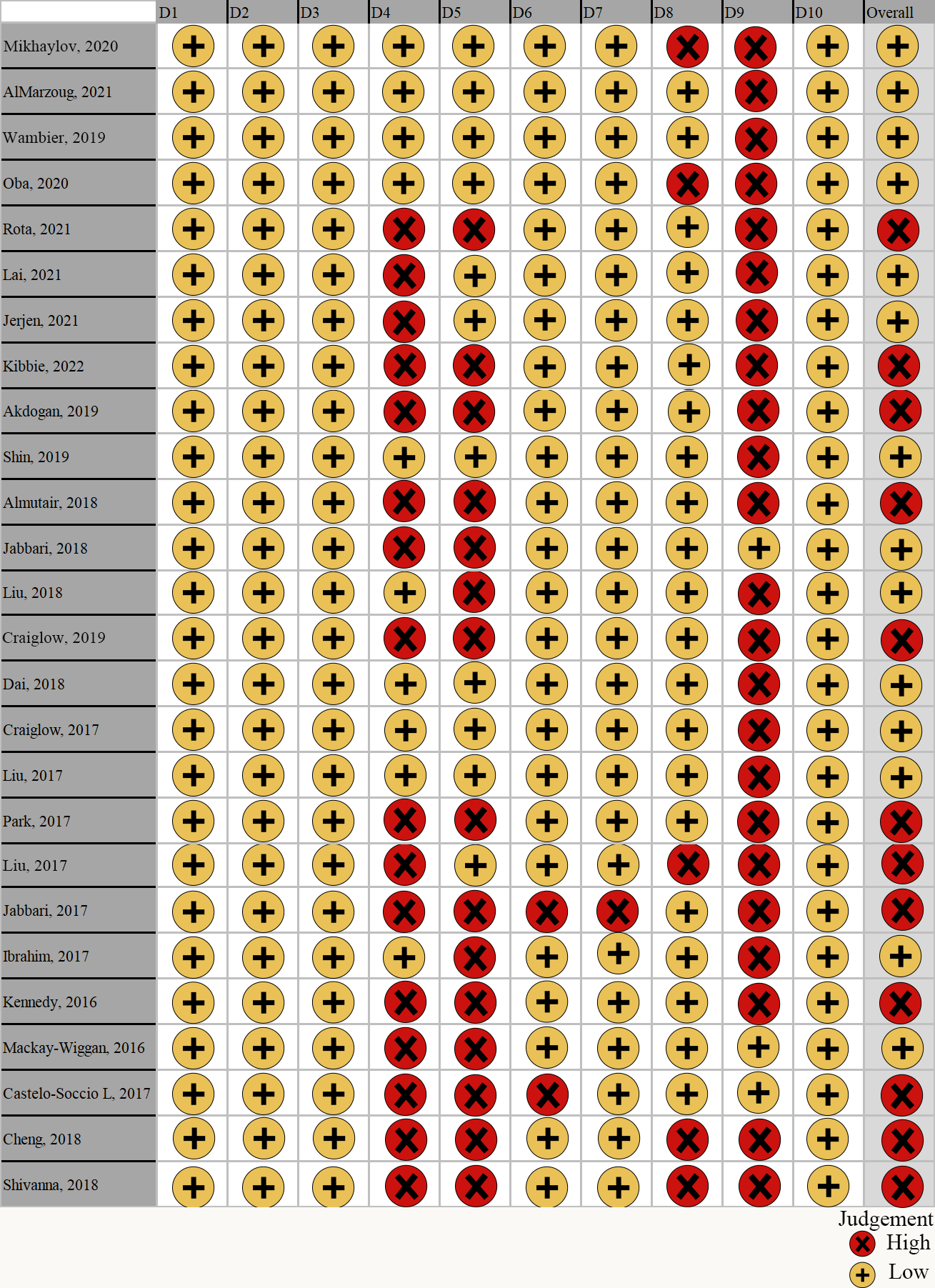


D1: Were there clear criteria for inclusion in the case series?

D2: Was the condition measured in a standard, reliable way for all participants included in the case series?

D3: Were valid methods used for identification of the condition for all participants included in the case series?

D4: Did the case series have consecutive inclusion of participants?

D5: Did the case series have complete inclusion of participants?

D6: Was there clear reporting of the demographics of the participants in the study?

D7: Was there clear reporting of clinical information of the participants?

D8: Were the outcomes or follow-up results of cases clearly reported?

D9: Was there clear reporting of the presenting sites’/clinics’ demographic information?

D10: Was statistical analysis appropriate?

**Mendeley Supplemental Figure 4.** Forest plot of the hair growth rate of JAKi treatment in randomized clinical trials. (A) Forest plot SALT_50_ rate compared to the placebo. (B) Forest plot SALT_90_ rate compared to the placebo. CI: Confidence interval. JAKi: Janus kinase inhibitor; SALT_50_: 50% improvement in Severity in Alopecia Tool; SALT_90_: 90% improvement in Severity in Alopecia Tool.


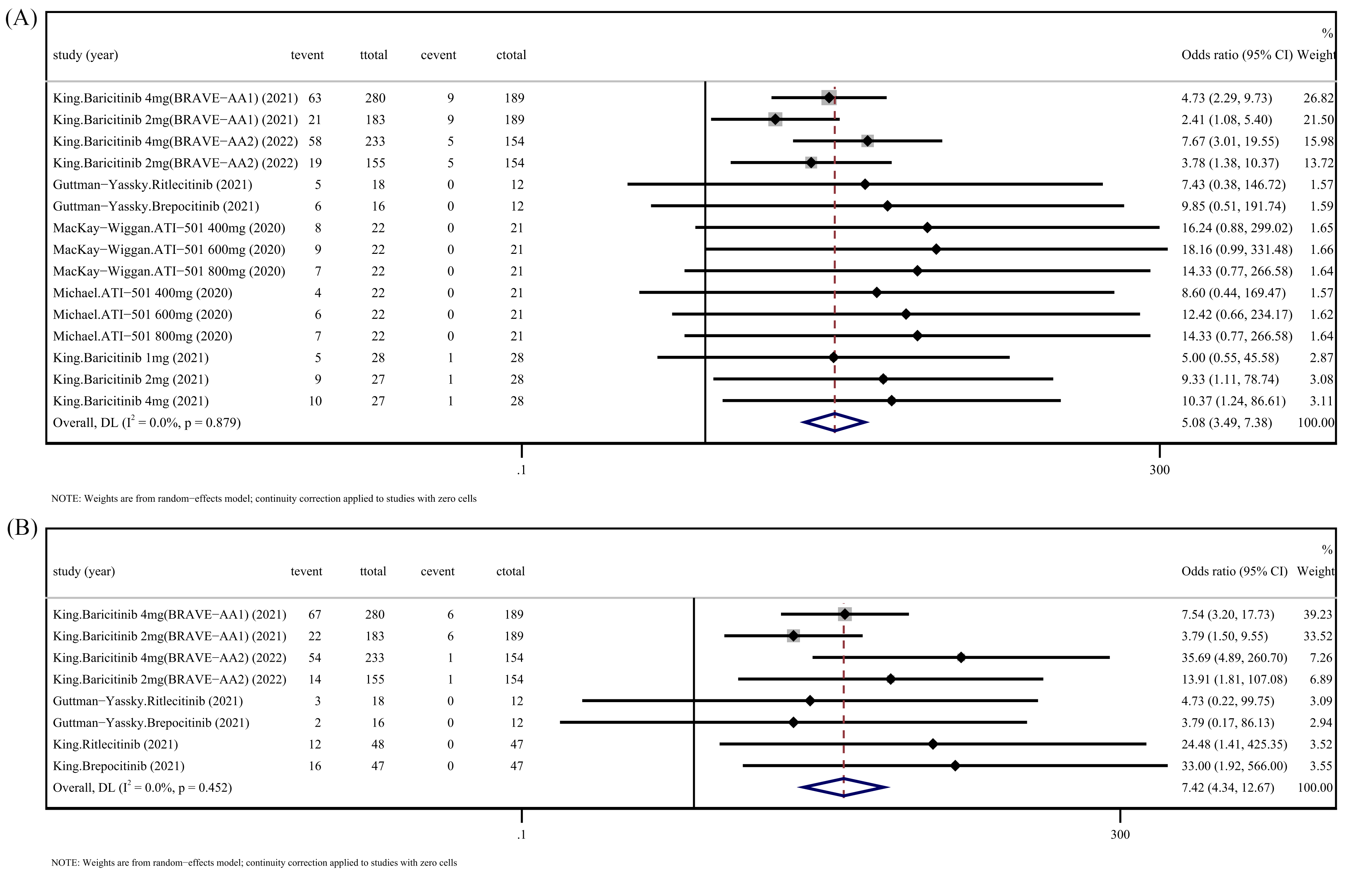


**Mendeley Supplemental Figure 5.** Forest plot of SALT_5_(A), SALT_50_(B), SALT_90_(C) rate of JAK inhibitors treatment in patients with AA in observational studies. SALT_5_: 5% improvement in Severity in Alopecia Tool; SALT_50_: 50% improvement in Severity in Alopecia Tool; SALT_90_: 90% improvement in Severity in Alopecia Tool.


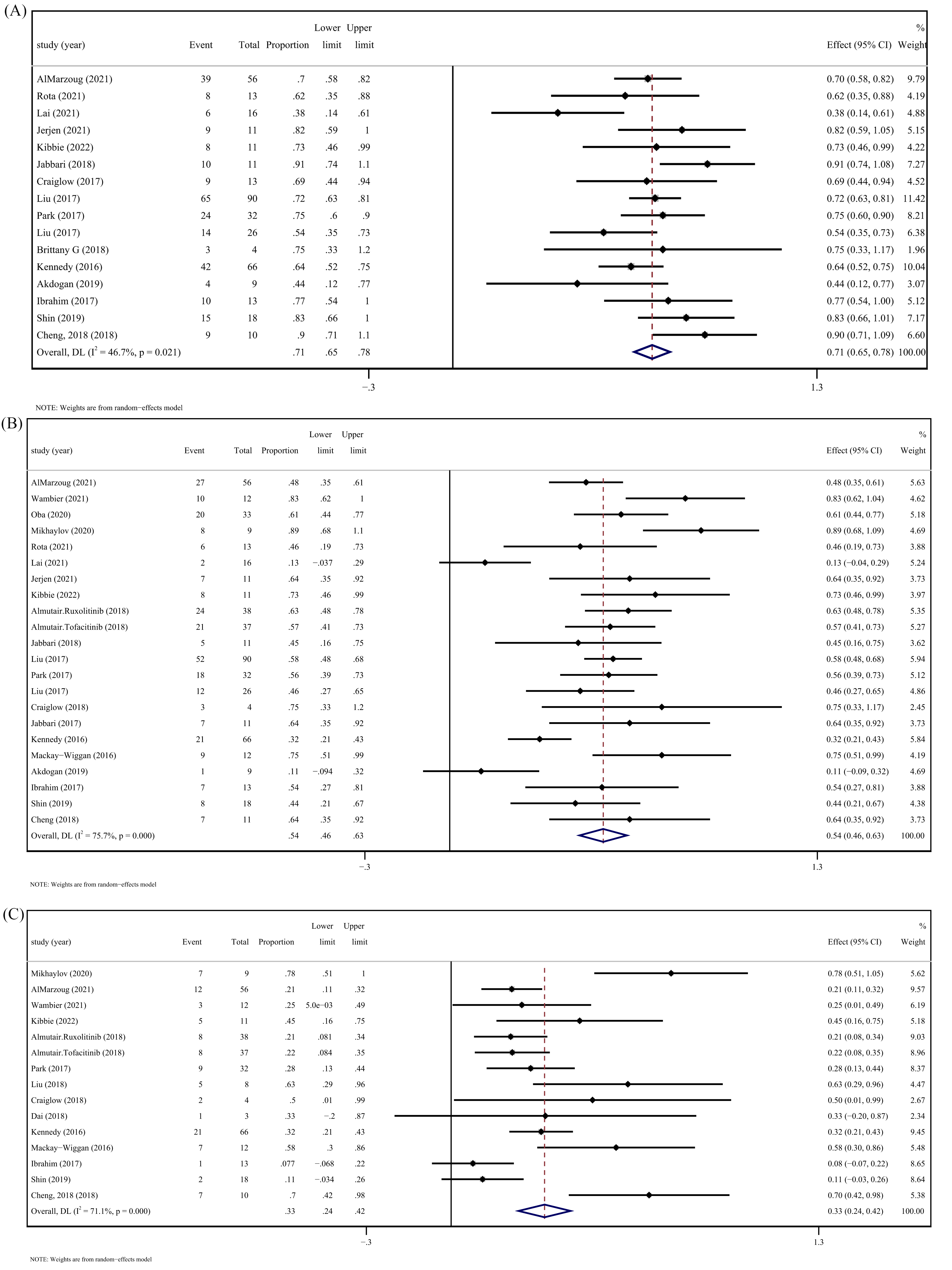


**Mendeley Supplemental Figure 6.** Pooled relative risk of total infection, stratified by drugs.


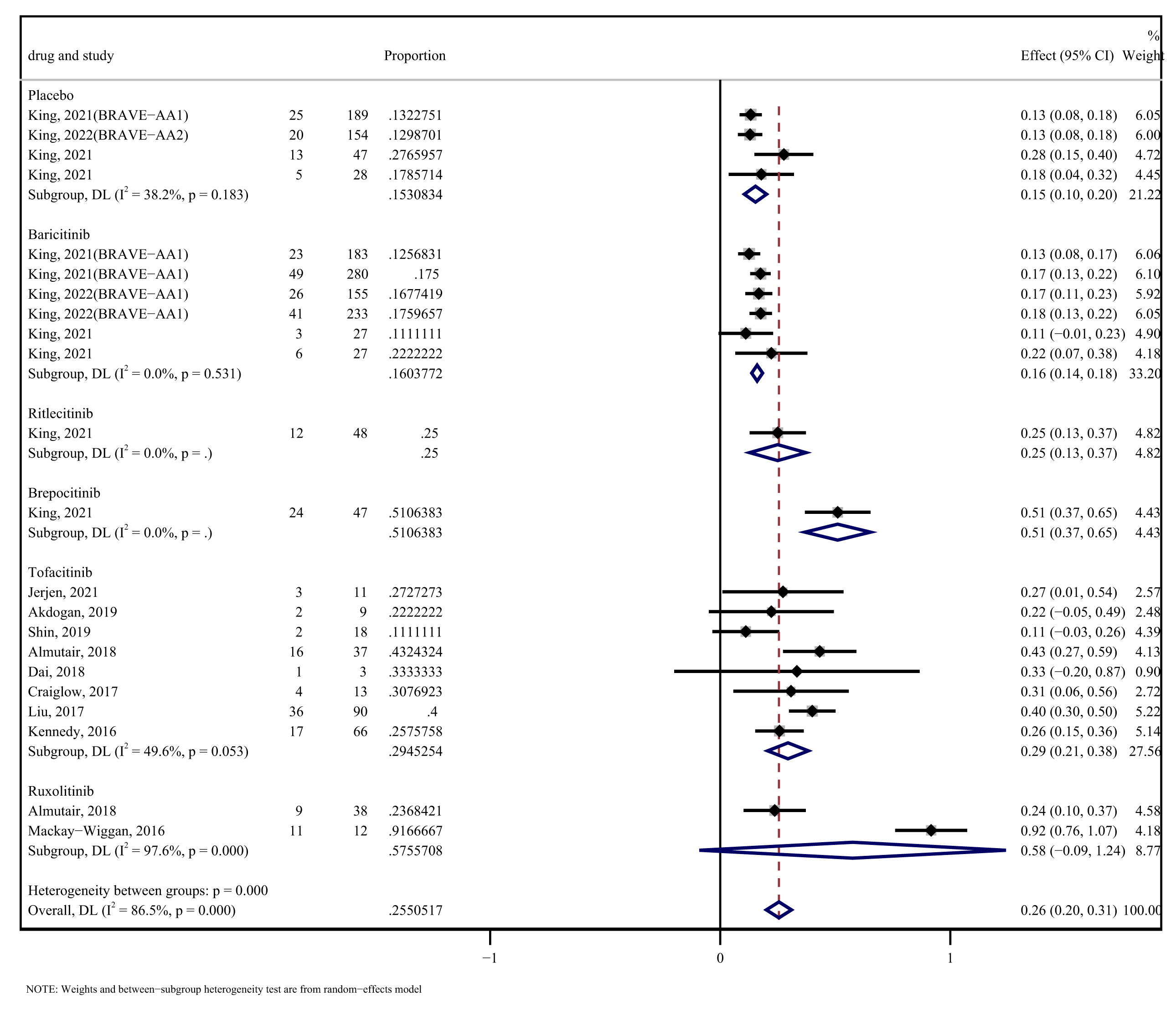


**Mendeley Supplemental Figure 7.** Pooled relative risk of laboratory abnormalities, stratified by drugs.


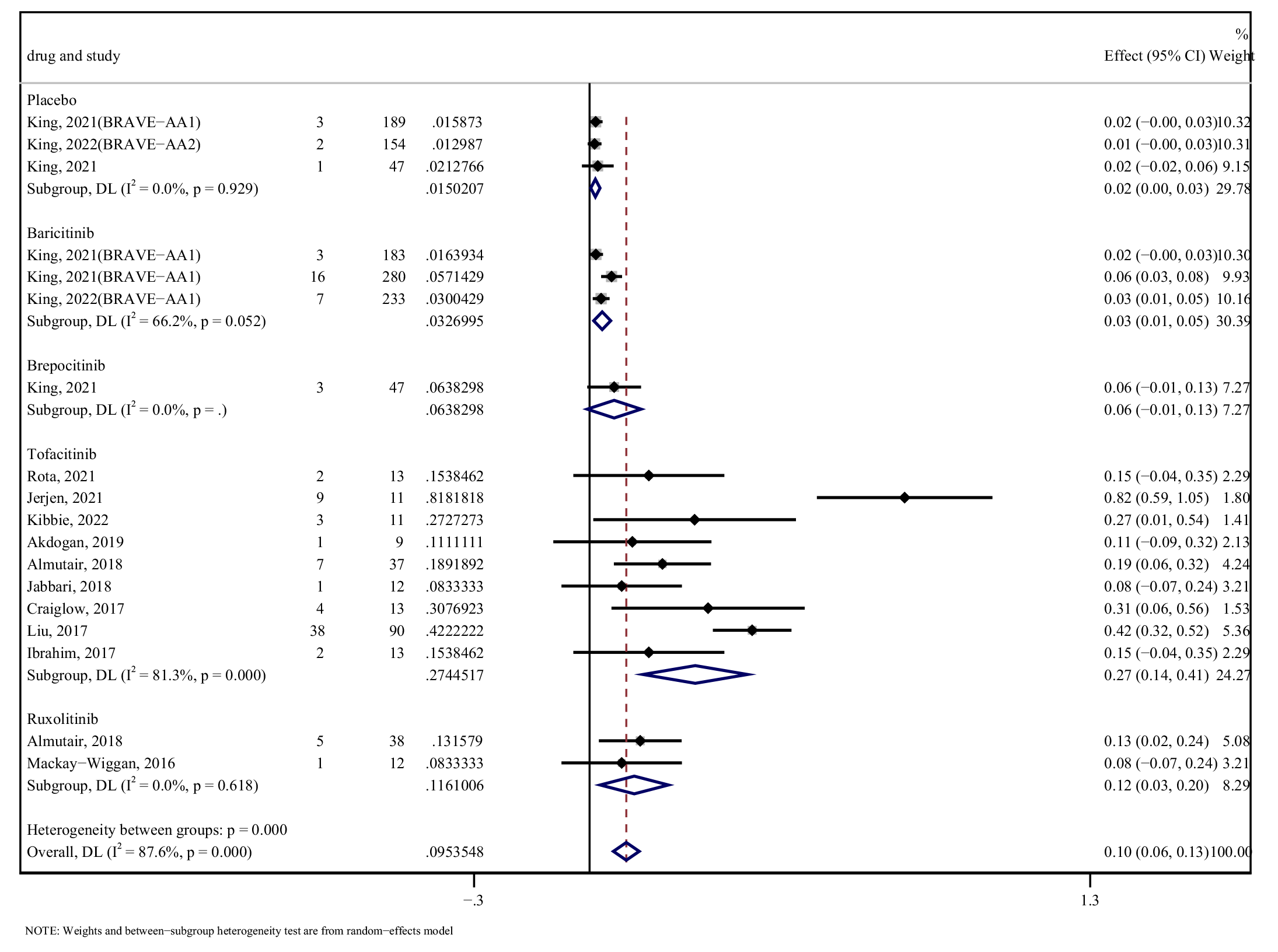


**Mendeley Supplemental Figure 8.** Pooled relative risk of neurological symptoms, stratified by drugs.


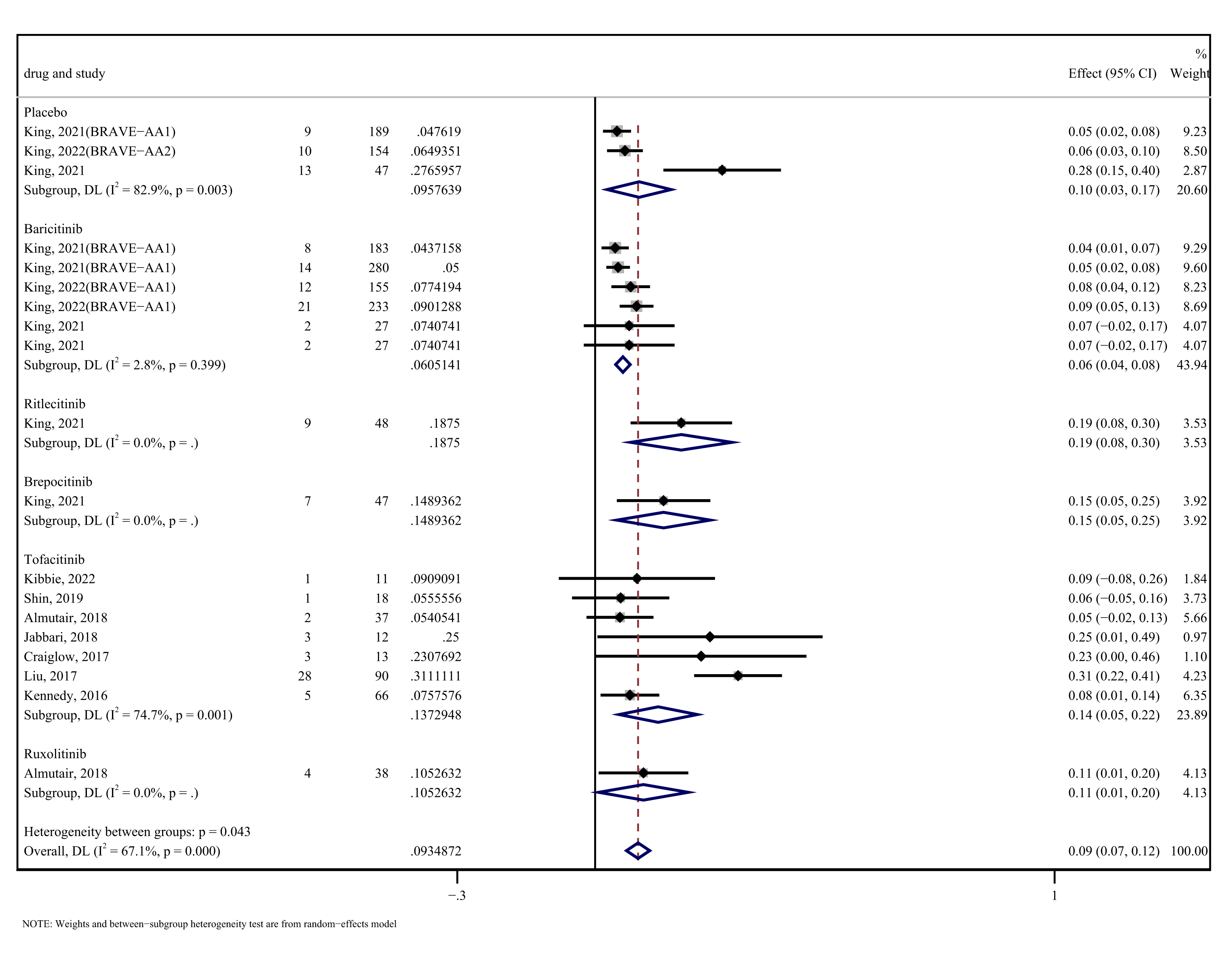


**Mendeley Supplemental Figure 9.** Pooled relative risk of gastrointestinal symptoms or weight gain, stratified by drugs.


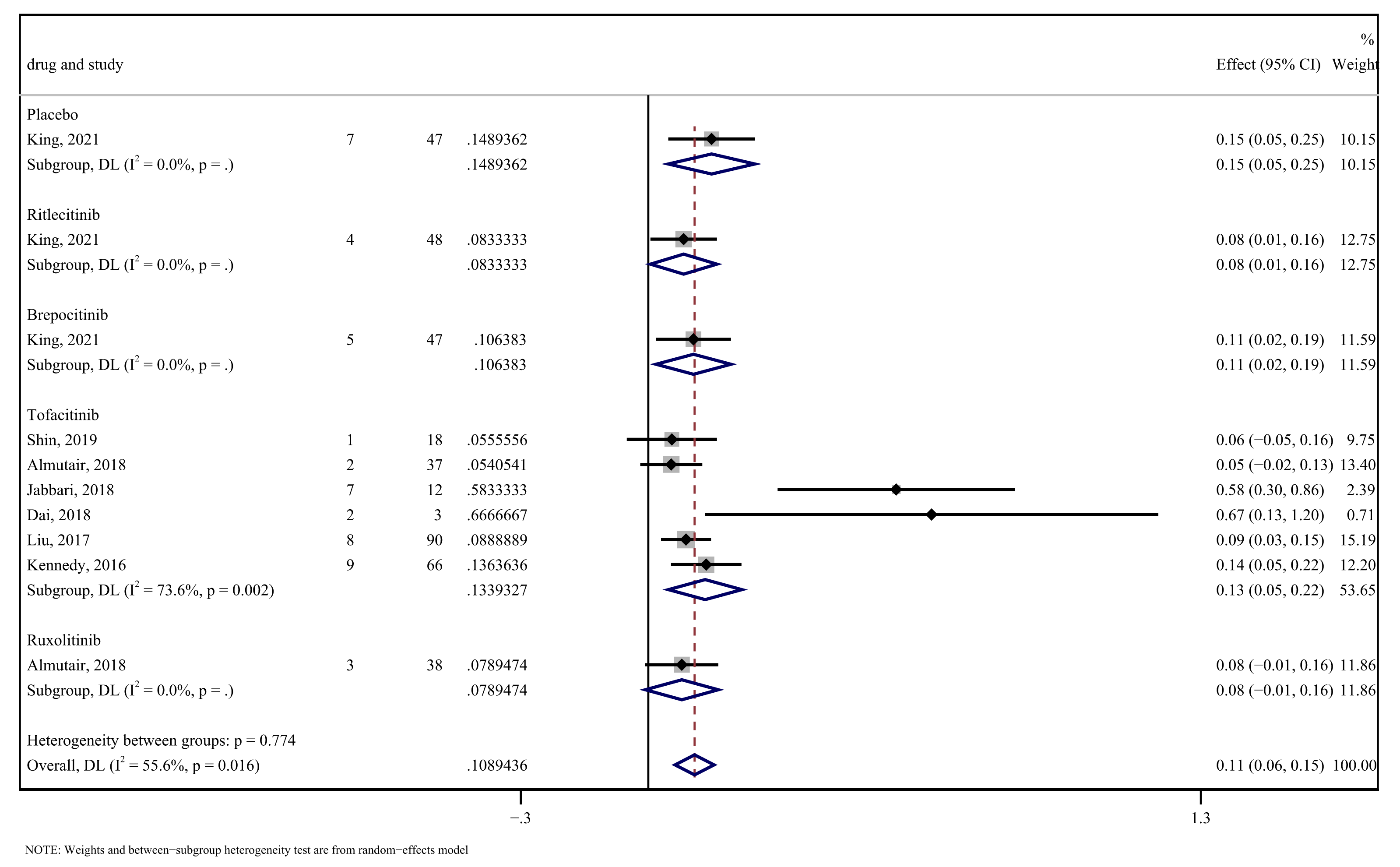


**Mendeley Supplemental Figure 10.** Pooled relative risk of cutaneous symptoms, stratified by drugs.


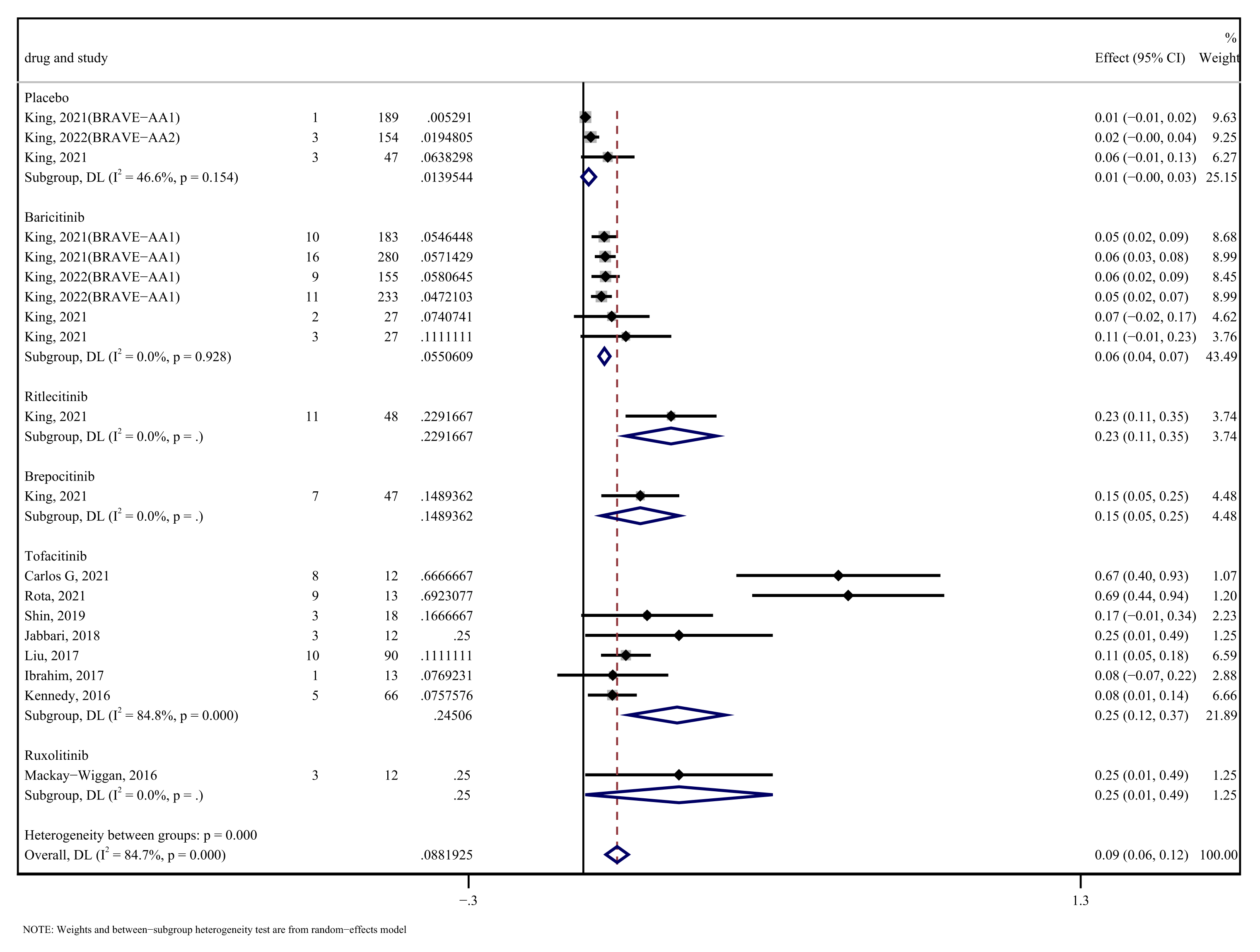


**Mendeley Supplemental Figure 11.** Begg’s test of SALT_50_ in randomized controlled trials.

**
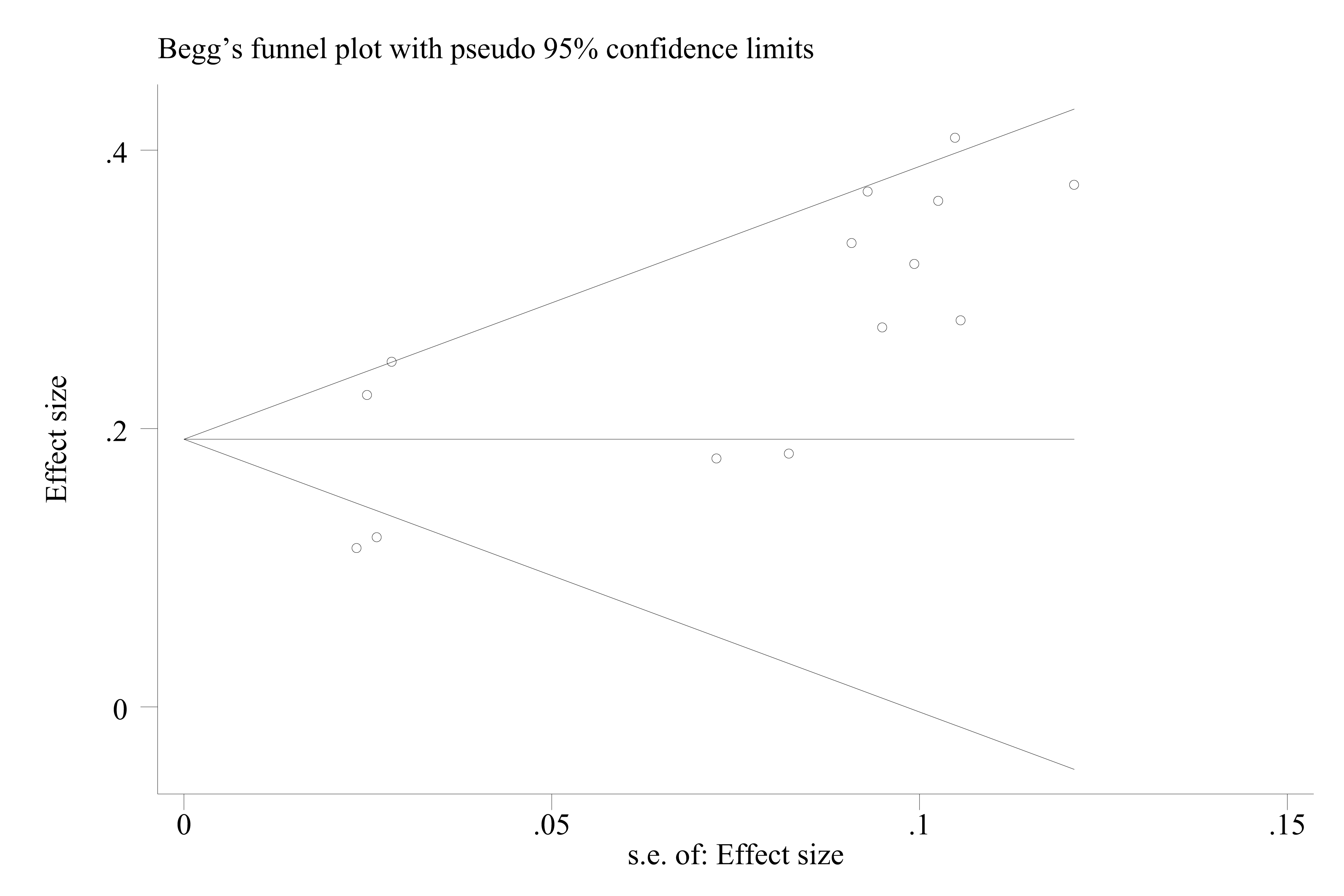
**

**Mendeley Supplemental Figure 12.** Begg’s test of SALT_50_ in non-randomized controlled trials.


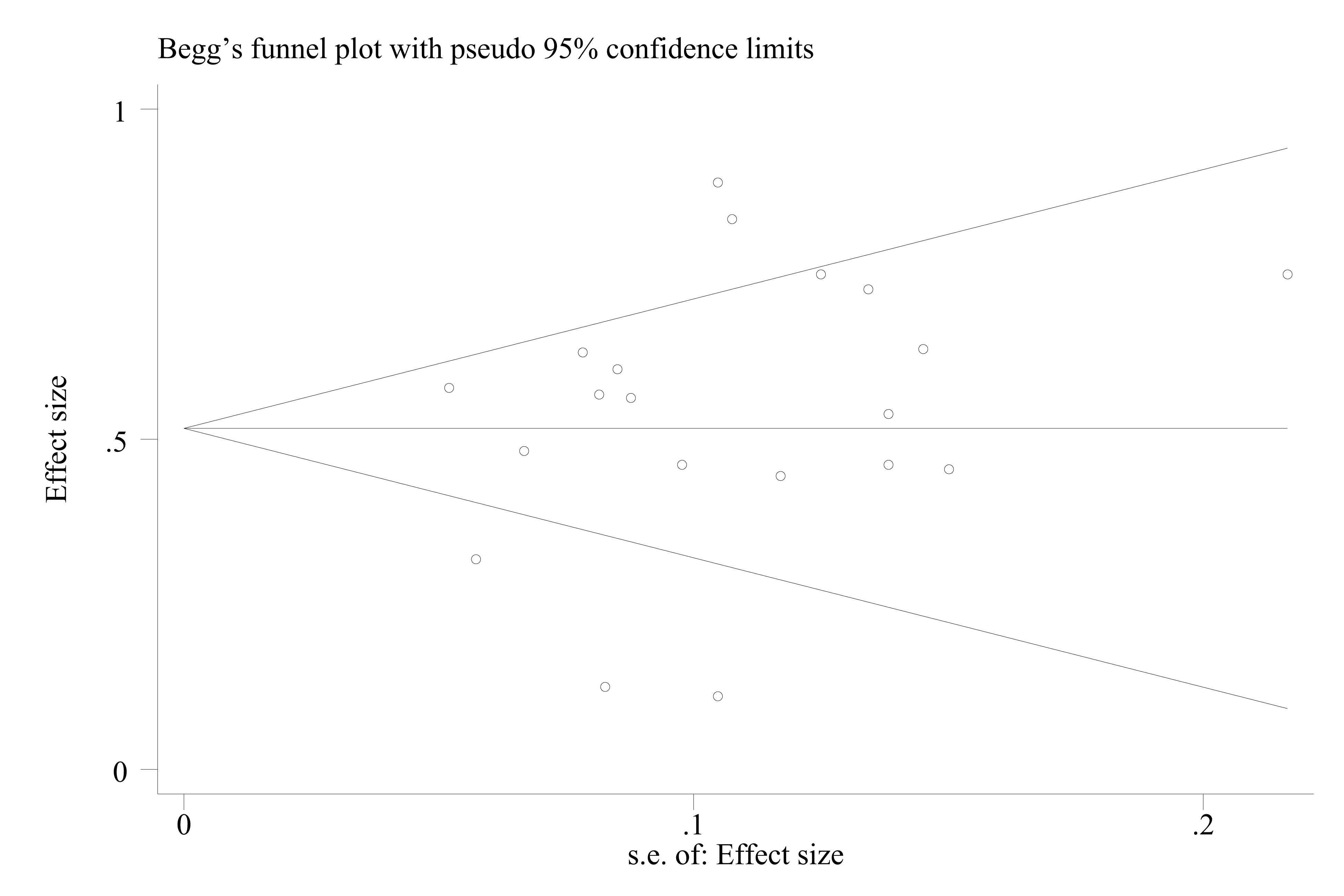


**Mendeley Supplemental Figure 13.** Funnel plot of SALT_50_ in non-randomized controlled trials.


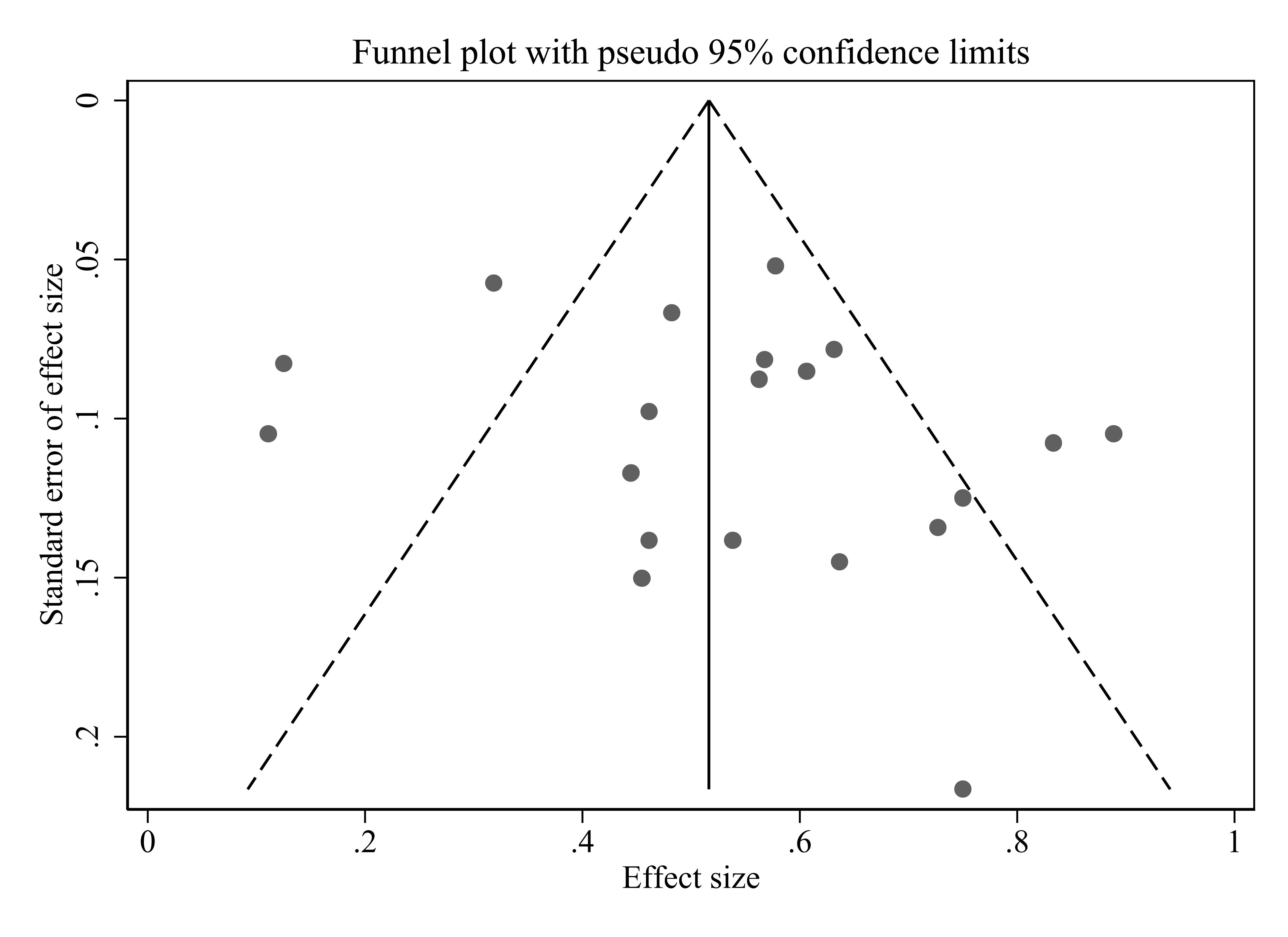

Supplement: Supplementary file 1 [file DataSheet_1.docx]
